# Extracellular vesicles from vaginal *Gardnerella vaginalis* and *Mobiluncus mulieris* contain distinct proteomic cargo and induce inflammatory pathways

**DOI:** 10.1038/s41522-024-00502-y

**Published:** 2024-03-21

**Authors:** Andrea Joseph, Lauren Anton, Yuxia Guan, Briana Ferguson, Isabella Mirro, Nova Meng, Michael France, Jacques Ravel, Michal A. Elovitz

**Affiliations:** 1 Women’s Biomedical Research Institute, Icahn School of Medicine at Mount Sinai, New York, New York, 10029 USA; 2 Department of Obstetrics, Gynecology and Reproductive Sciences, Icahn School of Medicine at Mount Sinai, New York, New York, 10029 USA; 3 Center for Research on Reproduction and Women’s Health, University of Pennsylvania, Philadelphia, PA 19104 USA; 4 Institute for Genome Sciences, University of Maryland School of Medicine, Baltimore, MD 21201 USA; 5 Department of Microbiology and Immunology, University of Maryland School of Medicine, Baltimore, MD 21201 USA

**Keywords:** Cellular microbiology, Clinical microbiology

## Abstract

Colonization of the vaginal space with bacteria such as *Gardnerella vaginalis* and *Mobiluncus mulieris* is associated with increased risk for STIs, bacterial vaginosis, and preterm birth, while *Lactobacillus crispatus* is associated with optimal reproductive health. Although host-microbe interactions are hypothesized to contribute to reproductive health and disease, the bacterial mediators that are critical to this response remain unclear. Bacterial extracellular vesicles (bEVs) are proposed to participate in host-microbe communication by providing protection of bacterial cargo, delivery to intracellular targets, and ultimately induction of immune responses from the host. We evaluated the proteome of bEVs produced in vitro from *G. vaginalis*, *M. mulieris*, and *L. crispatus*, identifying specific proteins of immunologic interest. We found that bEVs from each bacterial species internalize within cervical and vaginal epithelial cells, and that epithelial and immune cells express a multi-cytokine response when exposed to bEVs from *G. vaginalis* and *M. mulieris* but not *L. crispatus*. Further, we demonstrate that the inflammatory response induced by *G. vaginalis* and *M. mulieris* bEVs is TLR2-specific. Our results provide evidence that vaginal bacteria communicate with host cells through secreted bEVs, revealing a mechanism by which bacteria lead to adverse reproductive outcomes associated with inflammation. Elucidating host-microbe interactions in the cervicovaginal space will provide further insight into the mechanisms contributing to microbiome-mediated adverse outcomes and may reveal new therapeutic targets.

## Introduction

Vaginal microbial communities are associated with a spectrum of outcomes in gynecological and reproductive health. When dominated by *Lactobacillus*, vaginal communities are rich with lactic acid, antimicrobial substances, immunomodulatory compounds, and other bacterial factors that are believed to protect against sexually transmitted infection (STI), bacterial vaginosis (BV), and spontaneous preterm birth^1–3^. In contrast, in *Lactobacillus*-deficient communities, a diverse array of strict and facultative anaerobes deteriorates epithelial integrity by producing mucin-degrading enzymes, cytolysins, and other pro-inflammatory compounds^4–6^. These communities often include *Gardnerella vaginalis* and species of *Mobiluncus*, *Prevotella*, and *Sneathia*. High-diversity, *Lactobacillus*-deficient, anaerobe-dominated vaginal microbial communities have been associated with increased risk for many adverse reproductive outcomes including BV, HIV, spontaneous preterm birth, endometriosis, and infertility^7–11^. A common anaerobic bacteria implicated in these adverse outcomes is *G. vaginalis* with its within-species high genomic diversity and pathogenic potential^8–11^. Another associated species, identified by recent work from our laboratory using a large pregnancy cohort, is the anaerobic bacteria *Mobiluncus curtisii/ mulieris*^12^. Despite our decades-long understanding that anaerobic vaginal bacteria confer risk of poor reproductive outcomes, the precise mechanisms and host-microbe interactions driving these adverse outcomes have remained elusive.

An emerging body of literature provides evidence that diverse set of bacteria produce extracellular vesicles (EVs)^13,14^. Similar to eukaryotic EVs, bacterial EVs (bEVs) facilitate the transfer of biomolecules between cells and have been implicated in bacteria-bacteria and bacteria-host interactions including antibiotic resistance, biofilm formation, quorum sensing, regulation of host immunity, and maintenance of epithelial integrity^15^. Although the role of bEVs in the reproductive tract has been less studied, recent work has suggested that bEVs may contribute to reproductive health and disease^16–21^. We now hypothesize that bEVs from common vaginal anaerobes have discrete effects in the lower reproductive tract and thus are drivers of adverse reproductive outcomes.

In this study, we sought to characterize and functionally assess bEVs produced by *Lactobacillus crispatus* as a representative microbe associated with optimal reproductive outcomes, and *G. vaginalis* and *M. mulieris* as anaerobic bacteria associated with adverse reproductive outcomes. Our objectives were to (1) characterize in vitro-produced bEVs by morphology and proteomic analysis, (2) assess internalization of bEVs by vaginal and cervical epithelial cells, (3) determine the ability of bEVs to induce immune responses in epithelial and immune cells, and (4) evaluate the role of TLR2 in bEV-induced immune responses in epithelial cells.

## Results

### *L. crispatus*, *G. vaginalis*, and *M. mulieris* produce extracellular vesicles

**Fig. 1:**
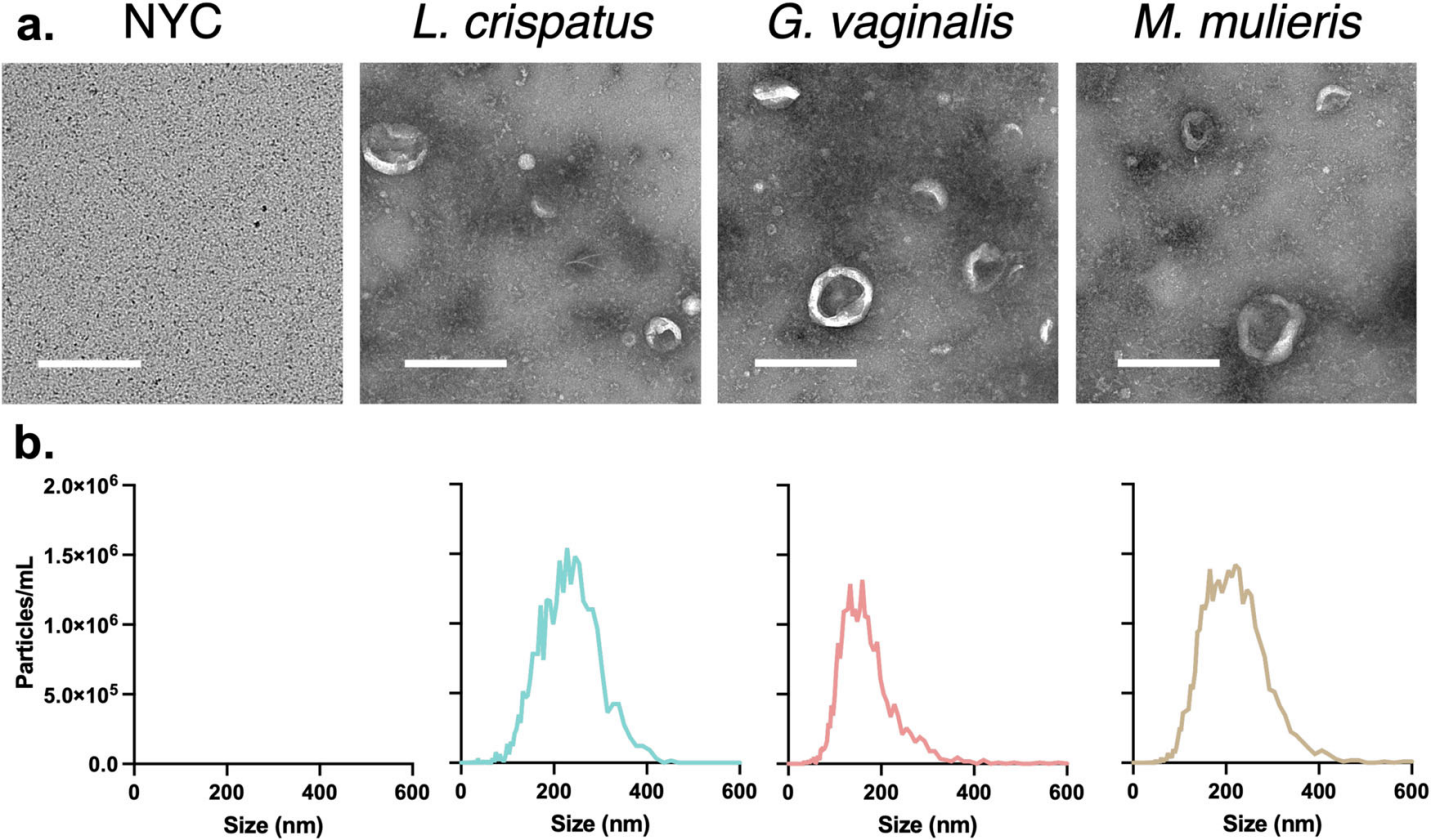
Size, morphology, and concentration of extracellular vesicles derived from *L. crispatus*, *G. vaginalis*, and *M. mulieris* grown in NYC culture medium. **a** Transmission electron microscopy indicates small, spherical vesicles isolated from bacterial culture samples but not NYC culture medium. **b** Particle size distributions from Nanoparticle Tracking Analysis indicate the presence of vesicles between 90 and 420 nm in diameter. All scale bars are 500 nm.

Bacterial EVs were isolated from *L. crispatus, G. vaginalis*, and *M. mulieris* culture supernatants using differential ultracentrifugation as previously described^16,17,21–23^. Transmission electron micrographs indicate the presence of spherical and cup-shaped structures of varying sizes (Fig. 1a). Images of the NYC culture medium alone show no such structures, indicating no contaminating vesicles of non-bacterial origin (Fig. 1a). These results were confirmed by ZetaView which indicated a nanoparticle size range of 90–420 nm in diameter for all bacterial samples (Fig. 1b). The mean ± standard deviation vesicle diameter was 159.5 ± 61.7 nm, 146.0 ± 50.9 nm, and 156.4 ± 55.0 nm for *L. crispatus*, *G. vaginalis*, and *M. mulieris* isolates, respectively.

### bEVs from L. crispatus, G. vaginalis, and M. mulieris contain distinct protein cargos

**Fig. 2:**
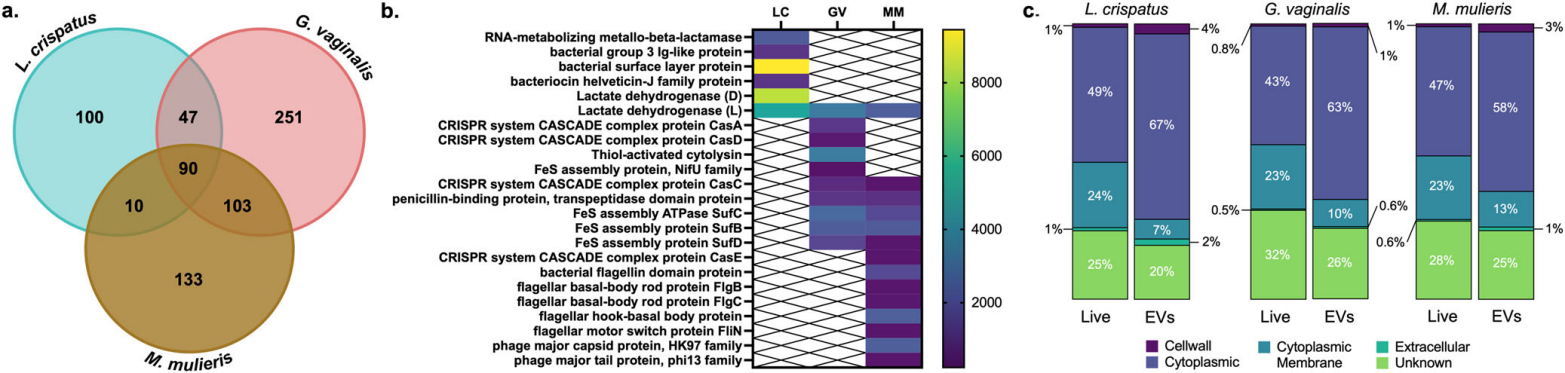
Proteomic analysis of extracellular vesicles derived from *L. crispatus*, *G. vaginalis*, and *M. mulieris* grown in NYC culture medium. **a** Out of 1074 total bacterial proteins identified, 90 orthologs were shared between all three species’ isolates, and 247, 491, and 336 were specific to *L. crispatus*, *G. vaginalis*, and *M. mulieris* isolates, respectively. **b** Select shared and distinct proteins are of functional interest; protein abundance (proteins per million) is shown by heat map. **c** Proteins were classified according to predicted subcellular localization.

Confirming production of bEVs from *L. crispatus, G. vaginalis*, and *M. mulieris*, we next sought to characterize their proteomic cargos. Gel electrophoresis showed the presence of distinct protein profiles between the three bEVs, and the absence of proteins in NYC culture medium (Supplemental Fig. 1). By liquid chromatography-mass spectrometry and peptide analysis by MaxQuant, we identified 1745 proteins across all samples, 650 and 21 of which originated from contaminants from horse (a component of NYC media) and human sources, respectively. A total of 491, 336, and 247 proteins of bacterial origin were from *G. vaginalis*, from *M. mulieris*, and from *L. crispatus*, respectively (Fig. 2a). The portion of these proteins which had orthologous functions between bacterial species are totaled in the overlapping areas of Fig. 2a; the proteins in common to all three samples are listed individually in Supplemental Table 1. These shared proteins, including ribosomal proteins and metabolic enzymes, are consistent with reports of bEV proteomes from multiple species^16,19,24^.

Select proteins of functional interest, and their abundance in the bEV proteome (proteins per million, ppm), are indicated in Fig. 2b. Specific to bEVs from *M. mulieris* are flagellin-family proteins, including bacterial flagellin domain protein (1640 ppm), flagellar basal-body rod proteins FlgB (329 ppm) and FlgC (399 ppm), flagellar hook-basal body protein (2300 ppm), and flagellar motor switch protein FliN (329 ppm). We additionally identified two phage proteins, phage major capsid protein (2300 ppm) and phage major tail protein (329 pm), and one CRISPR-associated protein, CasE (329 pm), only present in *M. mulieris*-derived bEVs.

Specific to bEVs from *G. vaginalis* are vaginolysin (thiol-activated cytolysin, 3330 ppm), its most well-characterized virulence factor^5,25^, CasA (1160 ppm) and CasD (465 ppm), two CRISPR-associated proteins, and NifU (232 ppm), an iron-sulfur cluster assembly protein specifically involved in nitrogen-fixing catalysis. Another CRISPR-associated protein, CasC, is common between bEVs of *G. vaginalis* (852 ppm) and *M. mulieris* (328 ppm), as well as iron-sulfur cluster proteins SufB, SufC, and SufD (2250, 2630, and 1550 ppm in *G. vaginalis* EVs, respectively; 2140, 1640, and 329 ppm in *M. mulieris* EVs, respectively). Also shared by *G. vaginalis* and *M. mulieris* bEVs is penicillin-binding protein (1080 and 985 ppm, respectively) which can selectively bind penicillin and other β-lactam antibiotics to promote antibiotic resistance^26^.

Similarly, *L. crispatus* EVs contain RNA-metabolizing metallo-beta-lactamase, which can directly metabolize β-lactam antibiotics. *L. crispatus* bEVs also contained bacterial group 3 Ig-like protein (1090 ppm), bacterial surface layer protein (9440 pm), and bacteriocin helveticin-J family protein (1090 ppm), an antimicrobial compound active against other *Lactobacillus* species. Finally, although bEVs of each *L. crispatus*, *G. vaginalis*, and *M. mulieris* contain L-lactate dehydrogenase (5080, 3250, and 2300 ppm, respectively), only bEVs from *L. crispatus* contain D-lactate dehydrogenase (8350 ppm). A full list of bacterial proteins from each isolate and their abundance is given in Supplemental Tables 2–4.

We next categorized proteins based on predicted subcellular localizations (Fig. 2c). In the *L. crispatus* bEV proteome, 67%, 7%, 4%, and 2% of proteins were classified as cytoplasmic, cytoplasmic membrane, cell wall, or extracellular, respectively, in comparison to 49%, 24%, 1%, and 1% of proteins, respectively, in the predicted proteome of the whole cell. For *G. vaginalis* bEVs, 63%, 10%, 1%, and 0.6% of proteins were classified as cytoplasmic, cytoplasmic membrane, cell wall, or extracellular, respectively, in comparison to 43%, 23%, 0.8%, and 0.5% of proteins, respectively, in the predicted proteome of the whole cell. For *M. mulieris* bEVs, 58%, 13%, 3%, and 1% of proteins were classified as cytoplasmic, cytoplasmic membrane, cell wall, or extracellular, respectively, in comparison to 47%, 23%, 1%, and 0.6% of proteins, respectively, in the predicted proteome of the whole cell. Across the three bacterial species, bEVs are enriched in cytoplasmic proteins and, to a lesser extent, cell wall-associated proteins, while cell membrane-associated proteins are underrepresented relative to the whole bacterial proteome.

Finally, we categorized proteins based on predicted biological function (Table 1) similarly to previous reports^16,17,21^. *L. crispatus*, *G. vaginalis*, and *M. mulieris* bEVs each contained proteins associated with many metabolism- and replication-associated functions. Only *M. mulieris*-derived bEVs contained any proteins related to cytoskeleton function, and only *L. crispatus*-derived bEVs did not contain any proteins related to defense mechanisms or cell motility functions.

**Table 1:** Functional classification of proteins carried by bEVs derived from *L. crispatus*, *G. vaginalis*, and *M. mulieris* grown in NYC culture medium

Function	Proportion of proteome (%)			ANOVA (adjusted *p*-value)		
	*L. crispatus* (LC)	*G. vaginalis* (GV)	*M. mulieris* (MM)	LC-GV	LC-MM	GV-MM
Translation	23.22	17.92	15.39	<0.0001	0.0399	<0.0001
Carbohydrate transport & metabolism	11.73	9.97	15.24	ns	0.0114	<0.0001
Function unknown	13.06	8.29	9.69	ns	ns	ns
Amino acid transport & metabolism	10.76	8.41	7.69	ns	0.0228	ns
Nucleotide transport & metabolism	10.64	9.85	6.18	ns	<0.0001	<0.0001
Cell wall/ membrane	7.50	6.62	10.77	ns	ns	ns
Transcription	2.90	8.41	4.79	<0.0001	<0.0001	ns
Energy production & conversion	3.99	4.32	6.75	ns	<0.0001	0.0057
Coenzyme transport & metabolism	4.23	5.24	5.36	0.0285	0.0228	ns
Replication, recombination, and repair	1.57	8.41	1.69	<0.0001	ns	<0.0001
Posttranslational modification	2.78	3.75	4.72	ns	ns	ns
Inorganic ion transport & metabolism	1.21	3.11	3.59	0.0342	0.0171	ns
Signal transduction mechanisms	1.81	3.05	2.80	ns	0.57	ns
Lipid transport & metabolism	3.02	2.65	0.93	ns	ns	ns
Cell division	2.54	1.79	1.88	0.0228	0.0342	ns
Intracellular trafficking	0.73	1.21	1.79	ns	ns	ns
Cell motility	0.00	0.35	3.20	ns	<0.0001	<0.0001
Defense mechanisms	0.00	1.04	1.29	ns	ns	ns
Secondary metabolites biosynthesis, transport and catabolism	0.12	0.63	0.85	ns	ns	ns
Cytoskeleton	0.00	0.00	0.75	ns	0.0057	0.0057

### *L. crispatus*, *G. vaginalis*, and *M. mulieris* bEVs rapidly internalize within cervical and vaginal epithelial cells

**Fig. 3:**
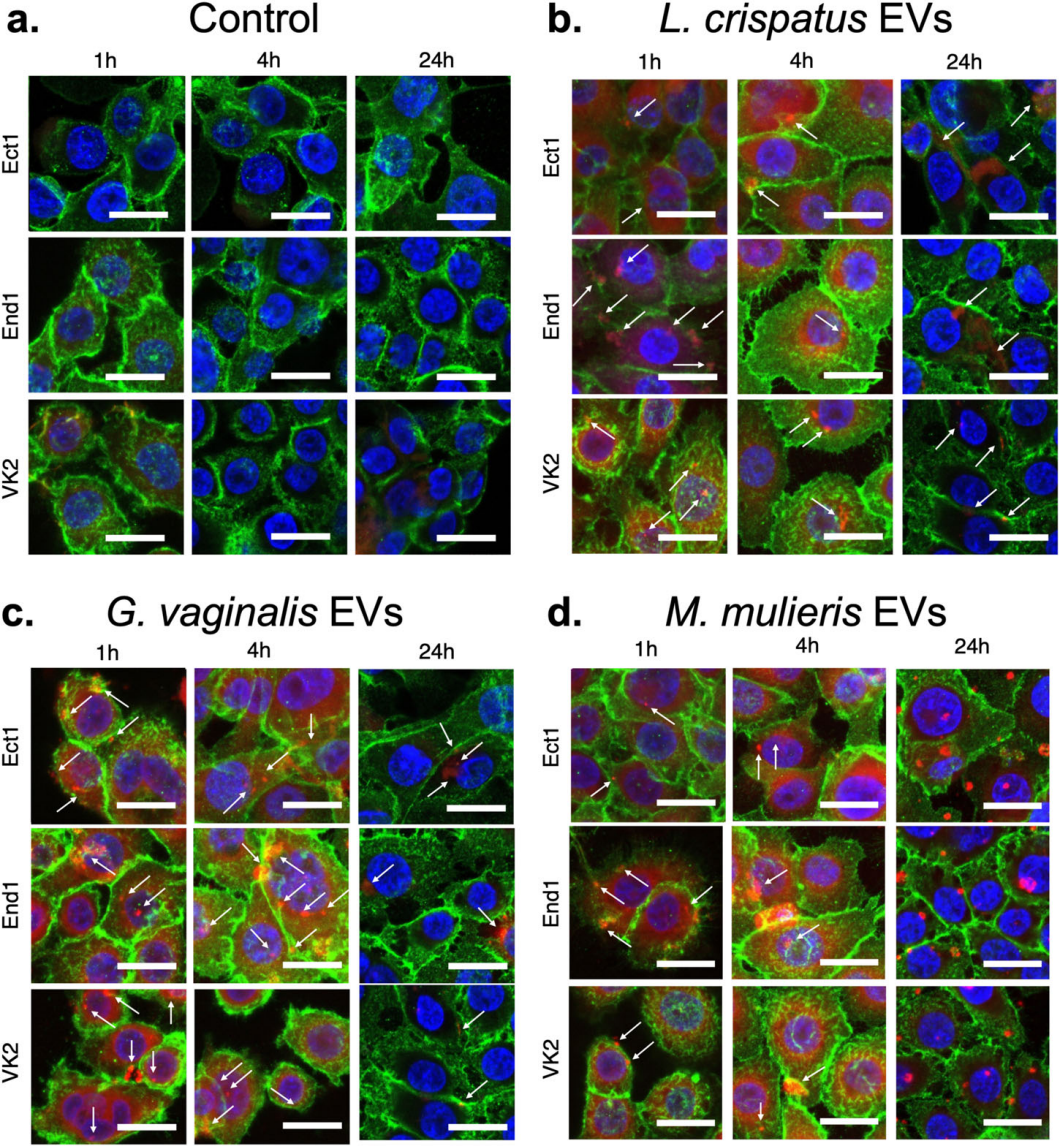
Uptake of *L. crispatus, G. vaginalis, and M. mulieris* bEVs by cervical and vaginal epithelial cells over 24 h. bEV preparations from (**a**) NYC culture medium (control), (**b**) *L. crispatus*, (**c**) *G. vaginalis*, and (**d**) *M. mulieris* were labeled with rhodamine B isothiocyanate and observed in the cytoplasm of ectocervical (Ect1), endocervical (End1), and vaginal epithelial (VK2) cells after 1, 4, and 24 h of incubation. All scale bars are 20 μm.

Having characterized the proteomic cargo of *L. crispatus*, *G. vaginalis*, and *M. mulieris* bEVs, we next determined whether these bEVs could be internalized in cervical and vaginal epithelial cells. Using confocal microscopy to visualize bEVs labeled with rhodamine B isothiocyanate (RBITC) and epithelial cells stained for E-cadherin, we observed cellular uptake of EVs after 1, 4, and 24 h of exposure to 10^9^ bEVs (equivalent to 5 × 10^3^ bEVs/cell, consistent with previous studies)^16,27^ (Fig. 3). At each timepoint assessed, bEVs from each vaginal bacteria were indicated by both punctate signal and diffuse fluorescence, suggesting the digestion of some bEVs and subsequent release of cell-permeant RBITC from the bEV interior to cell cytoplasm. No fluorescence in the red channel was observed after epithelial cell exposure to an equivalent concentration of RBITC alone at any timepoint (Fig. 3a). bEVs from *L. crispatus* were present at the cell surface of ectocervical, endocervical, and vaginal epithelial cells at 1 h, localized intracellularly by 4 h, and were largely cleared by 24 h (Fig. 3b). Similarly, bEVs from *G. vaginalis* were mostly observed at the cell interface at 1 h, within the cell by 4 h, and no longer abundant by 24 h (Fig. 3c). bEVs from *M. mulieris* were evident at the epithelial cell surface at 1 h but demonstrated an increased tendency to aggregate at the cell periphery at 4 h and distributed intracellularly by 24 h (Fig. 3d). When present intracellularly, bEVs from all three species appeared in cytoplasmic and perinuclear regions. Some differences in cell shape, like increased size and protrusions of the cell surface, were also observed at times of high bEV uptake. No differences in uptake between ectocervical, endocervical, and vaginal epithelial cell types were apparent in the time periods studied. Rapid endocytosis of bEVs by epithelial cells was confirmed by live imaging over 1 h for each bEV type and epithelial cell type (Supplemental Fig. 2 and Supplemental Videos 1-12).

### Cervical and vaginal epithelial cells produce bacterial-specific cytokine responses to bEVs

**Fig. 4:**
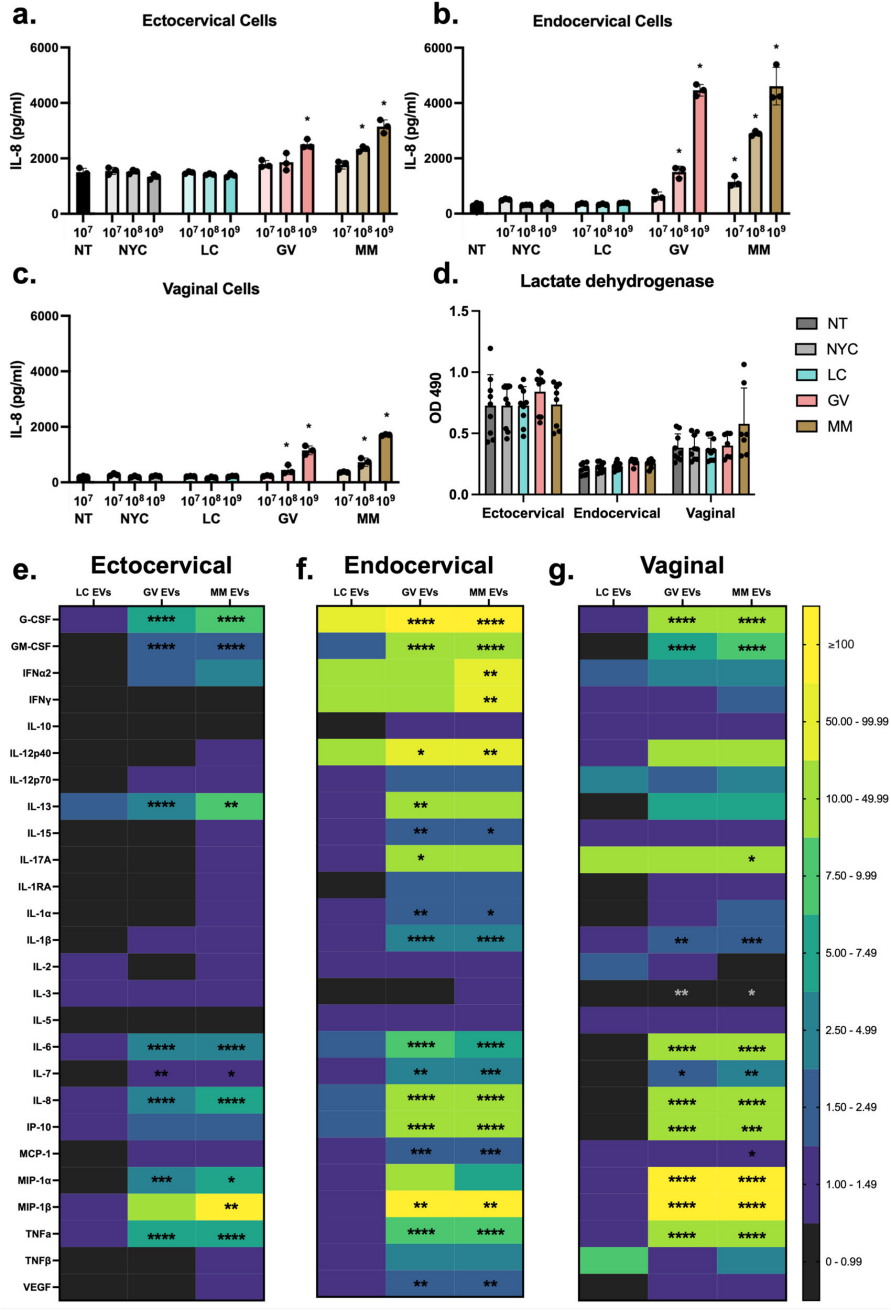
Dose-dependent cytokine responses from cervical and vaginal epithelial cells after 24 h exposure to *L. crispatus, G. vaginalis, and M. mulieris* bEVs. IL-8 expression measured by ELISA increases with increasing doses of bEVs from *G. vaginalis* and *M. mulieris*, but not *L. crispatus* and NYC culture medium (control), relative to nontreated (**a**) ectocervical, (**b**) endocervical, and (**c**) vaginal epithelial cells. **d** Exposure to bacterial EVs does not induce significant changes in lactate dehydrogenase release. 29-plex Luminex array identified a multi-cytokine response to bEVs from *G. vaginalis* and *M. mulieris*, but not *L. crispatus*, expressed as fold-changes relative to NYC culture medium control-treated (**e**) ectocervical, (**f**) endocervical, and (**g**) vaginal epithelial cells (*n* = 3 per treatment). Error bars indicate standard deviation and asterisks indicate adjusted *p*-values < 0.05 (*), < 0.01 (**), < 0.001 (***), and <0 .0001 (****) via one-way ANOVA with Tukey’s correction for multiple comparisons.

Understanding that vaginal and cervical epithelial cells can quickly internalize *L. crispatus, G. vaginalis*, and *M. mulieris* bEVs, we next sought to determine the immune responses induced by these bEVs from each cell type. We first assessed whether bEVs increase IL-8 expression in a dose-dependent manner. Cervical and vaginal epithelial cells (200,000 per well) were exposed to each bEV isolate at 10^7^, 10^8^, and 10^9^ bEVs/well for 1, 4, and 24 h. These doses are equivalent to 50, 500, and 5,000 bEVs/cell, which is consistent with previous work^27^. Cytokine responses were most robust after 24 h (Supplemental Fig. 3) and all subsequent assessments used this timepoint. In ectocervical cells at the highest dose of *G. vaginalis* bEVs, IL-8 expression increased 1.7-fold from non-treated (NT) cells. Exposure to the 10^8^ and 10^9^ doses of *M. mulieris* bEVs resulted in a 1.6- and 2.1-fold increase in IL-8 expression compared to NT, respectively. IL-8 expression was not significantly changed compared to NT after exposure to bEV preparations from NYC culture medium (control) and *L. crispatus* bEVs at any dose, *G. vaginalis* bEVs at the two lowest doses, and *M. mulieris* bEVs at the lowest dose (Fig. 4a).

A heightened dose-dependent response to *G. vaginalis* and *M. mulieris* bEVs was found in endocervical cells (Fig. 4b). Exposure to the 10^8^ and 10^9^ doses of *G. vaginalis* bEVs resulted in a 4.5- and 13.3-fold increase in IL-8 expression compared to NT, respectively. Doses of 10^7^, 10^8^, and 10^9^ *M. mulieris* bEVs led to a 3.4-, 8.6-, and 13.8-fold increase in IL-8 expression compared to NT, respectively.

The dose-dependent response to *G. vaginalis* and *M. mulieris* bEVs was similar in vaginal epithelial cells (Fig. 4c). The two highest doses of *G. vaginalis* bEVs led to a 2.1- and 5.6-fold increase in IL-8 expression from NT, respectively. Increasing doses of *M. mulieris* bEVs resulted in a 3.5- and 8.2-fold increase in IL-8 expression compared to NT, respectively. The observed changes in IL-8 expression were not due to bEV cytotoxicity and induction of cell death, as demonstrated by the lack of significant changes in lactate dehydrogenase release after 24 h of exposure to each bEV preparation at the highest dose (10^9^) across the three epithelial cell types (Fig. 4d).

To more comprehensively assess bEV-induced immune responses from cervical and vaginal epithelial cells, we conducted a 29-plex Luminex array on cell media after 24 h of exposure to each bEV preparation (Fig. 4e–g). Three analytes, EGF, eotaxin, and IL-4, were undetectable across all samples. For the remaining 26 cytokines, expression after exposure to bEVs preparation from NYC culture medium (control) was not significantly different compared to non-treated ectocervical, endocervical, or vaginal epithelial cells. Therefore, Fig. 4e–g shows fold-changes in cytokine expression after bEV exposure relative to NYC culture medium controls. In ectocervical cells, exposure to *G. vaginalis* bEVs resulted in significant increases of 8 cytokines: G-CSF, GM-CSF, IL-13, IL-6, IL-7, IL-8, MIP-1α, and TNFα (Fig. 4e). Ectocervical cell exposure to *M. mulieris* bEVs led to significant overexpression of 9 cytokines: G-CSF, GM-CSF, IL-13, IL-6, IL-7, IL-8, MIP-1α, MIP-1β, and TNFα (Fig. 4e). Exposure to *L. crispatus* bEVs did not result in significant changes in cytokine levels. The fold-change in cytokine expression and corresponding adjusted *p*-values, for each epithelial cell type, are listed in Supplemental Table 5.

In comparison to ectocervical cells, endocervical cells demonstrated a more pronounced immune response to *G. vaginalis* and *M. mulieris* bEVs, with each exposure inducing significantly increased levels of 16 cytokines (Fig. 4f) compared to controls. Fourteen of these cytokines were common to both *G. vaginalis* and *M. mulieris*: G-CSF, GM-CSF, IL-12p40, IL-15, IL-1α, IL-1β, IL-6, IL-7, IL-8, IP-10, MCP-1, MIP-1β, TNFα, and VEGF. Only *G. vaginalis* EVs resulted in a significant increase of IL-13 and IL-17A while only *M. mulieris* bEVs resulted in a significant increase in IFNα2 and IFNγ. Again, exposure to *L. crispatus* bEVs did not induce significant overexpression of any cytokine.

Vaginal epithelial cells also demonstrated a robust inflammatory response to *G. vaginalis* and *M. mulieris* bEVs, while no changes were observed with *L. crispatus* bEVs. Significantly altered levels of 11 and 13 cytokines were induced by *G. vaginalis* and *M. mulieris* bEVs, respectively (Fig. 4g). The 11 cytokines increased in response to *G. vaginalis* were also elevated after exposure to *M. mulieris*: G-CSF, GM-CSF, IL-1β, IL-3, IL-6, IL-7, IL-8, IP-10, MIP-1α, MIP-1β, and TNFα. Two cytokines were only altered by *M. mulieris* bEVs, IL-17A and MCP-1.

### *L. crispatus*, *G. vaginalis*, and *M. mulieris* bEVs induce immune responses from monocytes

**Fig. 5:**
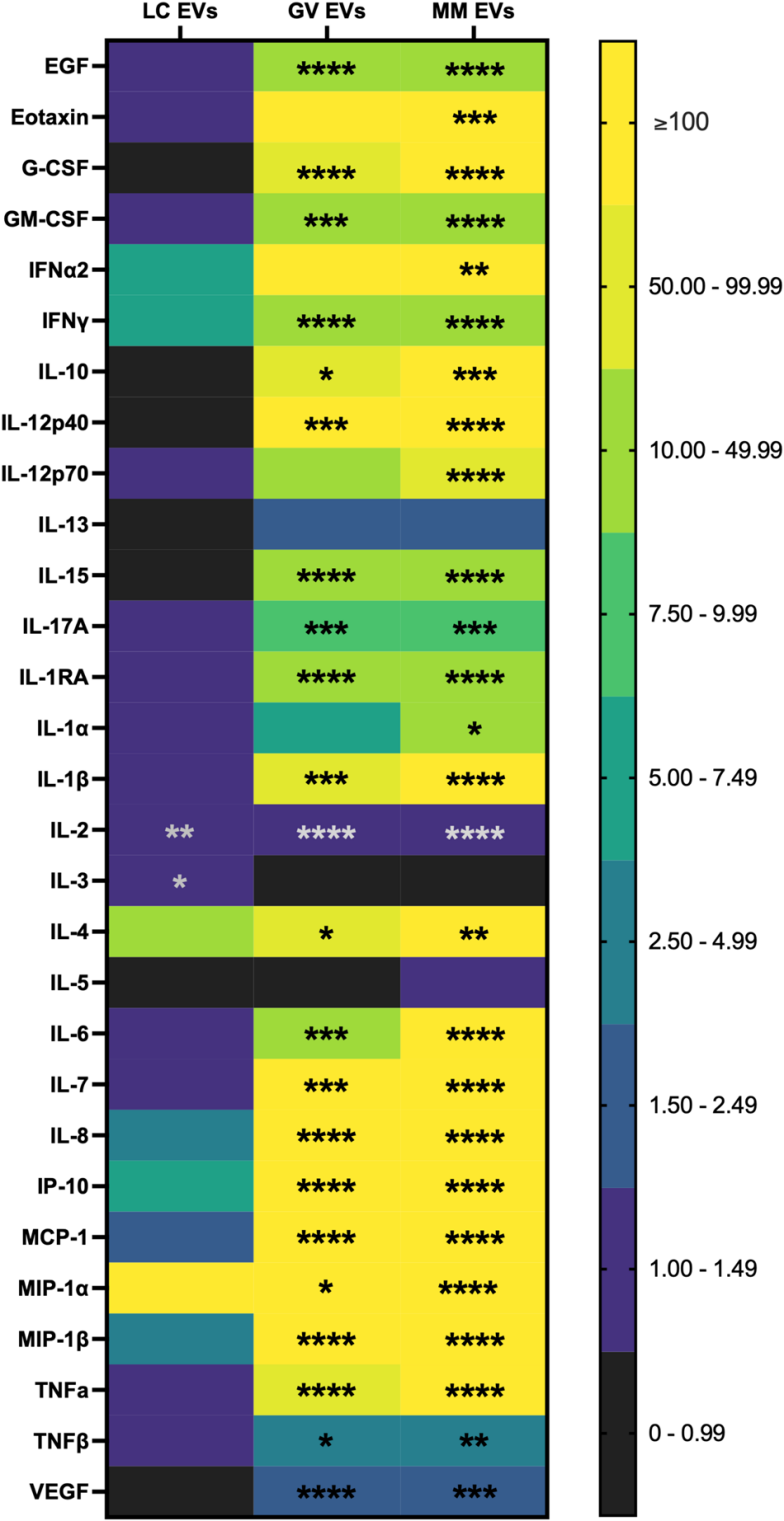
Cytokine responses from THP-1 monocytes after 24 h exposure to *L. crispatus, G. vaginalis, and M. mulieris* bEVs. 29-plex Luminex array identified a multi-cytokine response in THP-1 monocytes exposed to bEVs from *G. vaginalis* and *M. mulieris*, and a limited response to exposure to bEVs from *L. crispatus*, expressed as fold-changes relative to exposure to bEVs prepared from NYC culture medium (*n* = 3 per treatment). Error bars indicate standard deviation and asterisks indicate adjusted *p*-values < 0.05 (*), < 0.01 (**), < 0.001 (***), and < 0.0001 (****) via one-way ANOVA with Tukey’s correction for multiple comparisons.

Given that epithelial cells expressed a pro-inflammatory cytokine response to *G. vaginalis*- and *M. mulieris*-derived bEVs, we next assessed the ability of these bEVs to induce immune responses from immune cells common to the cervicovaginal space. Using THP-1 monocytes as a representative immune cell type^28–30^, we exposed cells to each *L. crispatus, G. vaginalis, and M. mulieris* bEV for 24 h and then analyzed cell culture media with the 29-plex Luminex cytokine/chemokine array, as was done with cervicovaginal epithelial cells, to investigate the activation of an innate immune response. No significant changes in cytokine levels were induced by bEV preparations from NYC culture medium (control) relative to non-treated monocytes. Exposure to bEVs from *L. crispatus* altered the levels of just 2 cytokines while bEVs from *G. vaginalis* and *M. mulieris* induced significant increases in 22 and 26 cytokines relative to NYC-treated cells, respectively (Fig. 5). All of the cytokines overexpressed after exposure to *G. vaginalis* were also increased by *M. mulieris* bEVs: EGF, G-CSF, GM-CSF, IFNγ, IL-10, IL-12p40, IL-15, IL-17A, IL-1RA, IL-1β, IL-2, IL-4, IL-6, IL-7, IL-8, IP-10, MCP-1, MIP-1α, MIP-1β, TNFα, TNFβ, and VEGF. Exposure to *M. mulieris* bEVs additionally increased expression of eotaxin (761-fold), IFNα2 (33.9-fold), IL-12p70 (54.3-fold), and IL-1α (10.6-fold). A full list of cytokine expression levels with fold-changes and adjusted *p*-values can be found in Supplemental Table 6.

### Cytokine response to L. crispatus, G. vaginalis, and M. mulieris bEVs is mediated through TLR2-activated signaling pathways

**Fig. 6:**
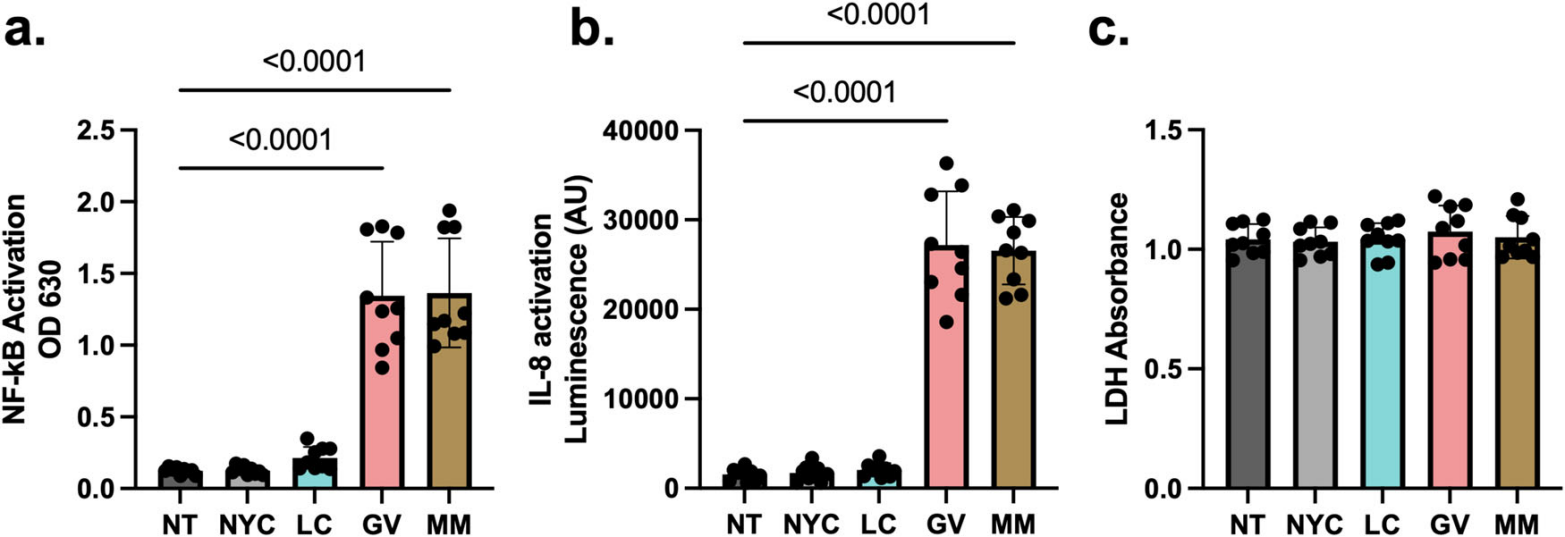
TLR2-specific pathways activated by *L. crispatus, G. vaginalis, and M. mulieris* bEVs. In HEK-TLR2 reporter cells exposed to bEVs for 24 h, *G. vaginalis* and *M. mulieris* bEVs induce significantly increased expression of (**a**) NF-kB and (**b**) IL-8. **c** Exposure to any bEV treatment does not induce cell death relative to non-treated controls, as measured by lactate dehydrogenase release. Error bars indicate standard deviation and *p*-values were calculated via one-way ANOVA with Tukey’s correction for multiple comparisons.

Studies from our lab and others have shown that *G. vaginalis* induces an innate immune response through TLR2^31,32^. Therefore, we sought to assess if the intracellular immune pathways activated by *L. crispatus, G. vaginalis, and M. mulieris* bEVs was dependent on TLR2 activation. Using HEK-TLR2 reporter cells, we found that only *G. vaginalis* and *M. mulieris* bEVs induced activation of NF-kB (Fig. 6a) and subsequent release of IL-8 (Fig. 6b) relative to non-treated cells. Both responses were found to be dose-dependent (Supplemental Fig. 4). In contrast, *L. crispatus* bEVs did not activate either NF-kB or IL-8 at any dose. Cell death was not significantly affected by treatment with any bEV types compared to non-treated cells (Fig. 6c).

## Discussion

In this study, we have comprehensively characterized bEVs derived from clinically relevant vaginal bacteria, i.e. *L. crispatus, G. vaginalis*, and *M. mulieris*. These bacteria are known to be associated with reproductive health and disease. bEVs from these vaginal bacteria carry potent bacterial cargo that are capable of internalizing within cervical and vaginal epithelial cells. Ascribing a novel mechanism by which vaginal anaerobes drive adverse reproductive outcomes, bEVs from *G. vaginalis* and *M. mulieris* induce immune responses from both epithelial and immune cells. Similarly, providing biological rationale for the association between *Lactobacillus* spp. and reproductive health, bEVs from *L. crispatus* do not induce immune activation in these same cell types. Collectively, our results provide new insights into the molecular mechanisms by which these vaginal bacteria can alter the cervicovaginal environment leading to diverse adverse reproductive outcomes.

Novel to this study, we demonstrated bEV production from *M. mulieris* and are among the first to describe bEV production from *L. crispatus* and *G. vaginalis*^16,17,19^. Similar to previous reports, we isolated bEVs by differential ultracentrifugation and confirmed the presence of bEVs from cultures of all three bacteria grown in vitro under optimized conditions using electron microscopy and nanoparticle tracking analysis. bEVs ranged in size from 90 to 420 nm in diameter, similar to EVs derived from eukaryotic cells and other bacteria^15^. This characteristic size suggests bEV biogenesis by blebbing of the inner membrane rather than explosive cell lysis, which results in EVs up to 800 nm in diameter and death of the originating cell^13,14,33^. In contrast, blebbing-derived cytoplasmic membrane vesicles are not dependent on cell death and in fact can promote bacterial survival through communication with bacterial and host cells^14,34–38^. Aligned with cytoplasmic biogenesis, proteomic analysis indicated that *L. crispatus, G. vaginalis*, and *M. mulieris* bEVs are enriched in cytoplasmic proteins relative to their live bacterial counterparts. While cytoplasmic proteins composed the majority of each bEV proteome, cell wall-, cell membrane-, and extracellular-associated proteins were also present in our analysis, similar to previous reports for Gram-positive bacteria including *Gardnerella* and *Lactobacillus* isolates^16,17^.

Several proteins of functional interest were identified by proteomic analysis, providing insight into mechanisms of microbiome-mediated pathogenicity and protection of the vaginal microenvironment. *G. vaginalis* bEVs contain vaginolysin, a pore-forming toxin capable of inducing cell lysis in cervicovaginal epithelial cells and red blood cells, and which is present in higher concentrations in women with indicators of bacterial vaginosis^25^. *M. mulieris*, a flagellated motile bacteria, produce bEVs that contain several flagellin-family proteins (flagellin domain protein, basal body rod proteins, hook-basal body protein, and motor switch protein) which may contribute to stimulation of an immune response mediated by TLR5^39^. Additionally, *M. mulieris* bEVs contain phage proteins (capsid and tail) which are capable of immune stimulation^40^. Both *G. vaginalis* and *M. mulieris* bEVs contain CRISPR-associated proteins (CasA, CasC, CasD, and CasE) which are responsible for DNA targeting; iron-cluster proteins (SufB, SufC, SufD, and NifU) which are critical to electron transport but can contribute to oxidative stress^41^; and penicillin-binding protein, which can bind penicillin and other β-lactam antibiotics to promote antibiotic resistance^26^. Comparisons of the bEV proteome across different bacterial species associated with adverse reproductive outcomes are novel and critical to further our understanding of the molecular pathways involved in microbe-host interactions in reproduction. These insights should inform future treatment strategies as the impact of bEVs appears to be associated with negative outcomes and likely worsened by current antibiotic therapeutics^42–46^.

Whereas proteins carried by *G. vaginalis* and *M. mulieris* bEVs are likely to promote a host inflammatory response, proteins in *L. crispatus* bEVs may serve to protect the epithelial barrier. Bacterial surface layer (S-layer) proteins, for example, mediate adherence to epithelial cells and protect against degradative enzymes in the mucus^47^. *L. crispatus* is the only known vaginal species to produce an S-layer which has been previously linked to high adherence to cervicovaginal epithelial cells and antagonism to pathogens of the genitourinary tract^48,49^. Incorporation of this protein into the *L. crispatus* bEVs suggests that there might be enhanced EV binding to epithelial cells, facilitating cellular internalization. Similarly, bacterial proteins with Ig-like domains (distinct from human antibodies) have many functional roles including adhesion. These proteins may be useful for facilitating bEV-cell contact and reducing motility of flagellated bacteria, protecting the vaginal microenvironment^50,51^. Lastly, bacteriocin helveticin-J family protein is an antimicrobial agent that inhibits the growth of closely related *Lactobacillus* species. Helveticin may contribute to *L. crispatus* regulation and dominance of the vaginal microbiome if bEVs are degraded extracellular or internalized by competing bacteria, which must be investigated in future studies^52^.

A major advantage of intercellular communication by bEVs compared to soluble factors is the ability to carry cargo to a destination cell. Here, we found that bEVs from each vaginal bacteria tested were internalized by vaginal (VK2), endocervical, and ectocervical epithelial cells within 1–4 h of exposure. While some previous studies have found VK2 uptake of live *L. crispatus*, *G. vaginalis*, and *G. vaginalis-*derived EVs within this time period, our study importantly adds the characterization of cervical epithelial cells^17,53^. Compared to vaginal epithelial cells, cervical epithelial cells have different embryological origins, cellular functions, and have previously demonstrated decreased responsiveness to microbial stimulation^32^. Despite different embryological origins, our findings support the ability of both cervical and vaginal cells to quickly internalize bEVs. Whether the mechanism of entry or the incorporation of bEV cargo is similar between the different epithelial cell lines requires further investigation. Further studies must investigate cervicovaginal mucus as a natural barrier to bEV uptake in vivo, although the small size and other biological properties of EVs may still enable cellular interaction. Inhibitors of endocytosis and cytoskeletal restructuring, such as cytochalasin-D, or of bacterial EV production, such as indole and amidine, are additional potential therapeutic strategies to prevent or treat the effects of EV uptake by cervical and vaginal epithelial cells^53,54^.

One main function of cervical and vaginal epithelial cells is to recruit an inflammatory response to bacterial threats. Several previous studies have shown a robust multi-cytokine response to live *G. vaginalis* and other BV-associated bacteria in vaginal, endocervical, and ectocervical epithelial cells, although the number of elevated cytokines and their increase in expression varies widely between bacteria and epithelial cell type^55–57^. Our laboratory has also previously found that culture supernatants from *G. vaginalis* and *M. mulieris* can recapitulate the pro-inflammatory response in the three epithelial cell lines, partially dependent on TLR2 activation and signaling^32,58^. In the present study, we observed robust inflammatory signaling from each epithelial cell line upon exposure to *G. vaginalis* and *M. mulieris* bEVs but not *L. crispatus* bEVs. In contrast to prior experiments with live and supernatant *G. vaginalis* exposure which found vaginal epithelial cells to have the most potent inflammatory response, our study found that endocervical cells were the most sensitive responders to bEVs^32^. This result reinforces the specificity of epithelial cells and bacterial product in mediating host-microbe interactions in the reproductive tract.

While epithelial cells in the reproductive tract can contribute to local inflammation, resident immune cells also play a critical role in immune regulation in the cervicovaginal space. Demonstrating the potential role of immune cells in host-microbe interactions, we show that stimulation of monocytes by *G. vaginalis* and *M. mulieris* bEVs induced a potent cytokine response; this response in monocytes was increased relative to epithelial cells at the same bEVs/cell ratio and nearly every measured cytokine was significantly increased after 24 hours of bEVs exposure. THP-1 cells have been previously used to study immune reactions to cervicovaginal bacteria^27,59,60^. In a previous study, exposure of THP-1 cells to live *G. vaginalis* resulted in cell death, production of cytokines and reactive oxygen species, and markers of NLRP3 inflammasome-mediated pyroptosis^60^. In another study, *L. crispatus* induced differentiation of THP-1 cells into a dendritic-like phenotype^59^. Our study adds to prior work by revealing how bEVs from common vaginal bacteria, and not just the live bacteria, can activate local immune cells. Our findings support the role of bEVs from *L. crispatus, G. vaginalis*, and *M. mulieris*, and certainly other vaginal bacteria, in mediating host-microbe interactions specifically by driving a pro-inflammatory response.

While there are likely diverse mechanisms by which vaginal bacteria produce and release bEVs and by which these bEVs induce molecular effects in cells, this study does demonstrate an important role for TLR2 signaling in the observed cytokine response to *G. vaginalis* and *M. mulieris* EVs but not to *L. crispatus* EVs. We have previously shown that live *L. crispatus* and *G. vaginalis* and their supernatants activate TLR2 signaling^32^. TLR2 is activated by bacterial components including lipoproteins, which are ubiquitous to all bacteria and highly expressed in the cytoplasmic membrane of Gram-positive bacteria including *L. crispatus*, *G. vaginalis*, and *M. mulieris*; of note, the latter two bacteria usually stain Gram-negative^31^. While all of these vaginal bacteria have a Gram-positive cell wall, lipoproteins were only found in the proteome of *G. vaginalis* and *M. mulieris* EVs, not *L. crispatus* EVs. As such, our proteomic findings are consistent with our in vitro findings that bEVs from *L. crispatus* do not activate TLR2. Therefore, bEVs represent an important mechanism by which *L. crispatus* can evade immune recognition by host cells, and by which host cells recognize and respond to many non-optimal vaginal bacteria.

Our study is limited by the use of ATCC, rather than clinical, strains of the selected vaginal bacteria. The selected ATCC strains of *G. vaginalis* and *M. mulieris* were originally isolated from women with bacterial vaginosis^61,62^, and each selected strain shares a high degree of genetic similarity with other clinical strains of the same species^63–65^. While the selected ATCC strain of *L. crispatus* has previously been shown to produce a high amount of lactic acid and be protective against *Chlamydia trachomatis*^66^, even phylogenetically closely related strains of *L. crispatus* have been shown to differ in production of lactic acid, bacteriocins, and other antimicrobial compounds^67^. Recent characterization of multiple clinical strains of *G. vaginalis* and even species of *Gardnerella* has shown marked variation in virulence properties (biofilm formation, sialidase activity, and antibiotic resistance), which may be linked to clinical outcomes^68^. Further study of the bEVs produced by clinical strains directly associated with adverse outcomes would provide insight into strain-specific differences in bacterial function and effects on reproductive outcomes. Additionally, our study only assesses three vaginal bacterial species associated with health and disease. While *G. vaginalis* and *M. mulieris* have been widely implicated in adverse reproductive outcomes, further studies will need to address whether additional non-optimal species (*Prevotella, Sneathia*) produce bEVs with functional importance; whether biogenesis, cargo, and functionality of bEVs are altered in polymicrobial states; and whether more complex models of the cervicovaginal epithelium and resident immune cell populations, especially models that include mucus, would reveal additional functional activities of bEVs. Metabolomic, RNA, and DNA cargo of bEVs must also be evaluated. Such investigation is currently technically challenging but would reveal bEV biology at the molecular scale and potentially lead to novel therapeutic strategies. Finally, recent results have demonstrated the ability of maternal gut microbiota-derived EVs to reach the fetal environment; analogously, whether vaginal bEVs can travel to the uterus and/or fetus must be studied^24^.

Overall, our study demonstrates that *L. crispatus, G. vaginalis*, and *M. mulieris* produce bEVs that carry specific proteomic cargo, are internalized within cervical and vaginal epithelial cells, and induce immune responses from epithelial and immune cells. bEVs represent a biological modulator for cellular crosstalk and for protected delivery of unstable cargo like proteins and genetic material to distant cells and tissues. Our results suggest that proteins from *G. vaginalis* and *M. mulieris* are delivered to host epithelial and immune cells by bEVs, inducing inflammation and potentially contributing to adverse reproductive outcomes. In contrast, several proteins from *L. crispatus* bEVs may confer protection to the host epithelium. This study provides a novel contribution by directly comparing the physical, biochemical, and immunogenic properties of bEVs from different vaginal bacterial species, enabling greater insight into protective and pathogenic host-microbe interactions in the vaginal environment. Further studies must evaluate bEVs as contributors to microbiome-mediated adverse outcomes and may reveal new therapeutic targets in the female reproductive tract.

## Methods

### Cell culture

Ectocervical (Ect/E6E7, ATCC CRL-2614), endocervical (End1/E6E7, ATCC CRL-2615), and vaginal (VK2/E6E7, ATCC CRL-2616) human epithelial cell lines (American Type Culture Collection, Manassas, VA) were cultured in keratinocyte-serum-free media (K-SFM) supplemented with 0.1 ng/mL epidermal growth factor and 50 μg/mL bovine pituitary extract (Gibco, Life Technologies), 100 U/mL penicillin, and 100 μg/mL of streptomycin at 37 °C in a 5% CO_2_ humidified incubator.

THP-1 monocytes were cultured in RPMI 1640 (Life Technologies, Grand Island, NY) supplemented with 10% fetal bovine serum (FBS), 10 mM HEPES, 0.1 mM MEM non-essential amino acids, 1 mM sodium pyruvate, 100 U/mL penicillin, and 100 μg/mL of streptomycin at 37 °C in a 5% CO_2_ humidified incubator.

TLR2 (NF-kB-SEAP/KI-IL-8 Lucia) dual-reporter human embryonic kidney (HEK) 293 cells (Invivogen, San Diego, CA), a TLR2 reporter cell line, express the human TLR2 gene, an NF-kB/AP1-inducible SEAP (secreted embryonic alkaline phosphatase) reporter gene, and the Lucia luciferase reporter gene under the control of the endogenous IL-8 promoter. These cells also show no activity of TLR3, TLR5, and TNFR (tumor necrosis factor receptor). HEK-TLR2 cells were grown in Dulbecco’s Modified Eagle’s Medium (DMEM, Mediatech, Corning, Glendale, AZ) containing 4.5 g/L glucose, 2 mM L-glutamine, 10% heat-inactivated FBS (30 min at 56 °C), 100 ug/mL Normocin (InvivoGen) and selective antibiotics 100 μg/mL Hygromycin B Gold (InvivoGen), and 50 μg/mL Zeocin (InvivoGen) at 37 °C in a 5% CO_2_ humidified incubator.

### Bacterial culture and isolation of extracellular vesicles

Bacteria were grown at 37 °C in an anaerobic glove box (Coy Labs, Grass Lake, MI). *G. vaginalis* (ATCC 14018*), L. crispatus* (ATCC 33197), and *M. mulieris* (ATCC 35243) were grown in New York City (NYC) III broth supplemented with 1% horse serum (Gibco, Thermo Fisher Scientific) and pre-cleared of extracellular vesicles by overnight ultracentrifugation at 100,000 x *g* and 4 °C. Bacterial growth was measured and quantified by colony forming unit (CFU) assays.

To remove bacterial cells and cell debris, cultures were centrifuged at 3500 x *g* for 30 min, filtered through a 0.04 μm filter (Fisher Scientific), and centrifuged again at 30,000 x *g* for 33 min. bEVs were then isolated from the cleared supernatant by ultracentrifugation at 100,000 x *g* for 70 min and washed once in phosphate buffered saline (PBS). Finally, the pellet containing bEVs was resuspended in 100 μL of 10 mM HEPES and 25 mM NaCl. Particle analysis and concentration assessment using ZetaView/ Nanoparticle Tracking Analysis (Particle Metrix) were performed with 2 μl of each sample. Samples were stored in −80 °C until use.

### Transmission electron microscopy

A 5 µL volume of sample was applied to a thin carbon grid that was glow discharged for 2 min using a Pelco Easyglow instrument. A 5 µL of freshly made 2% uranyl acetate stain solution was applied and incubated with the sample for 2 min on the grid. Excess sample and stain were blotted away with a Whatman filterpaper. The staining process was repeated and the grid was let to dry until imaged.

TEM micrographs were collected using Tecnai T12 TEM microscope at 100 KeV. The images were recorded on Gatan Oneview 4Kx4K camera. Each image was collected by exposing the sample for 4 s and a total of 100 dose fractionated images were collected and into a single micrograph. The data was collected at −1.5 to 2 microns under focus at 30 K–40 K magnification.

### Protein extraction

Samples were solubilized in 55 µL of extraction buffer containing 5% sodium dodecyl sulfate (SDS, Affymetrix), 8 M urea (Bio-Rad), 100 mM Tris-HCl pH 8.0 (Rockland), and protease inhibitor cocktail (Roche cOmplete, EDTA free). To shear DNA and ensure complete solubilization, samples were sonicated for 10 min at 10 °C in a Covaris R230 focused-ultrasonicator with the following settings: Dithering: Y = 3.0, Speed=20.0, PIP: 360.0, DF: 30, CPB: 200. Samples were centrifuged at 3000 x g for 10 min to clarify lysate. 1 µL of each sample was taken to estimate protein concentration by in-gel staining with Bradford Coomassie solution and intensity analysis with GelAnalyzer 19.1, using a serial dilution of an in-house generated *E. coli* lysate standard. All samples were processed in parallel from the same experiment.

### In-solution digestion

100 µg of each sample was digested per the S-Trap Micro (Protifi) manufacturer’s protocol^69^. Briefly, proteins were reduced in 5 mM TCEP (Thermo), alkylated in 20 mM iodoacetamide (Sigma), then acidified with phosphoric acid (Aldrich) to a final concentration of 1.2%. Samples were diluted with 90% methanol (Fisher) in 100 mM TEAB, then loaded onto an S-trap column and washed three times with 50/50 chloroform/methanol (Fisher) followed by three washes of 90% methanol in 100 mM TEAB. A 1:10 ratio (enzyme: protein) of Trypsin (Promega) and LysC (Wako) suspended in 20 µL 50 mM TEAB was added, and samples were digested for 1.5 hours at 47 °C in a humidity chamber. After incubation, peptides were eluted with an additional 40 μL of 50 mM TEAB, followed by 40 μL of 0.1% trifluoroacetic acid (TFA) (Pierce) in water, and finally 40 μL of 50/50 acetonitrile:water (Fisher) in 0.1% TFA. Eluates were combined and organic solvent was dried off via vacuum centrifugation. Samples were then desalted using an Oasis HLB µElution plate (30um, Waters). Wells were conditioned two times with 200 µL of acetonitrile and equilibrated three times with 200 µL of 0.1% TFA. Samples were applied, washed three times with 200 µL 0.1% TFA, and eluted directly into autosampler vials in three increments of 65 µL of 50:50 acetonitrile:water. Eluates were then dried by vacuum centrifugation and reconstituted in 0.1% TFA containing iRT peptides (Biognosys, Schlieren, Switzerland). Peptides were quantified with A280 measurement on a NanoDrop 1000 (Thermo) and adjusted to 0.4 µg/µL for injection.

### Mass spectrometry data acquisition

Samples were analyzed on a QExactive HF mass spectrometer (Thermofisher Scientific San Jose, CA) coupled with an Ultimate 3000 nano UPLC system and an EasySpray source. Peptides were separated by reverse phase (RP)-HPLC on Easy-Spray RSLC C18 2um 75 μm id × 50 cm column at 50 C. Mobile phase A consisted of 0.1% formic acid and mobile phase B of 0.1% formic acid/acetonitrile. Peptides were eluted into the mass spectrometer at 300 nL/min with each RP-LC run comprising a 95 min gradient from 1 to 3% B in 5 min, 3–45%B in 90 min. The mass spectrometer was set to repetitively scan m/z from 300 to 1400 (R = 120,000) followed by data-dependent MS/MS scans on the twenty most abundant ions, dynamic exclusion with a repeat count of 1, repeat duration of 30 s, (R = 15,000) and a nce of 27. FTMS full scan AGC target value was 3e6, while MSn AGC was 2e5, respectively. MSn injection time was 32 ms; microscans were set at one. Rejection of unassigned, 1, 6-8 and > 8 charge states was set.

### System suitability and quality control

The suitability of Q Exactive HF instrument was monitored using QuiC software (Biognosys, Schlieren, Switzerland) for the analysis of the spiked-in iRT peptides. Meanwhile, as a measure for quality control, we injected standard *E. coli* protein digest prior to and after injecting sample set and collected the data in the Data Dependent Acquisition (DDA) mode. The collected data were analyzed in MaxQuant and the output was subsequently visualized using the PTXQC package to track the quality of the instrumentation^70,71^.

### Protein identification and pathway analysis

The complete gene sequence of each peptide was retrieved by mapping (blastp, evalue < 10^−^^5^) to a database of each strain’s proteome. Proteomes were acquired from public databases (*G. vaginalis* ATCC 14018: GCF_003397685.1*, L. crispatus* ATCC 33197: ATTC Genome Portal, and *Mobiluncus mulieris* ATCC 35243: GCF_000160615.1. The identified complete gene sequences were annotated with function and localization predictions PSORTdb (v4.0, default settings)^72^, and eggNOG-mapper (v2.1.10, DB version 5.0.2, diamond v0.8.22, --evalue 0.001)^73^. To facilitate between species comparisons, orthologs were then identified among the three proteomes using OrthoFinder (v2.5.4, default settings)^74^.

### Fluorescent labeling, immunocytochemistry, and confocal imaging

Ectocervical, endocervical, and vaginal epithelial cells (*n* = 3 samples per condition) were plated at 2.0 × 10^5^ cells/well in 4-chamber slides (ibidi 80426) coated with 0.1% gelatin. bEVs were stained with rhodamine B isothiocyanate (RBITC, 0.2 mg/mL in 20 mM HCl) for 30 min, collected by centrifugation at 100,000 x *g* for 25 min, and washed once in PBS. Freshly stained bEVs (10^9^ bEVs/well) were added to the cells. Cells were fixed with 10% formalin at 1 h, 4 h, or 24 h and incubated with an E-cadherin primary antibody (ab231303, 1:50 dilution) and then secondary antibody (ab150105, 1:750 dilution) for 1 h at room temperature each. Cells were washed three times with cold 1x PBS for 5 min between steps. Slides were washed and dried for 30 min in the dark. Mounting medium (Dako, Agilent Technologies, Santa Clara, CA, USA) was added to each slide, and a glass coverslip was placed on top. Slides were stored at 4 °C (until imaged by Zeiss 880 confocal microscope in theCell & Developmental Biology Microscopy Core Facility) and at −20 °C for long-term storage.

In a subset of samples for live imaging, cells were plated at 2.0 × 10^5^ cells/dish on 35 mm high glass bottom μ-dishes (ibidi 81156) coated with 0.1% gelatin. After 24 h, Abcam Cytopainter staining solution (ab138891) was added to cells for 30 min and then washed three times in fresh media. Freshly stained bEVs (10^9^ bEVs/sample) were added to the cells. After another 30 min, cells were moved to a temperature-controlled chamber and imaged by the Zeiss 880 confocal microscope. At 5 independent locations per sample, 1 image was captured every minute for 30 min and compressed into a video file with 6 frames per second.

### Cell death, ELISA, and Luminex assays

Ectocervical, endocervical, vaginal, and THP-1 cells (*n* = 3 samples per condition) were plated at 2.0 × 10^5^ cells/well in 24-well plates containing cell media without antibiotics. The next day, the cells were treated with bEV preparations (10^9^ bEVs/well) from *L. crispatus*, *G. vaginalis, M. mulieris*, or NYC culture medium in cell medium for 24 h. At the end of each experiment, cell culture medium was collected for analysis of cell death using a lactate dehydrogenase (LDH) assay, IL-8 production by ELISA, or multiple cytokine expression by Luminex.

The release of lactate dehydrogenase (LDH) from cells (*n* = 3–9 independent experiments per cell type) was measured using the CytoTox 96 nonradioactive cytotoxicity assay (Promega, Madison, WI). Absorbance values were recorded from a colorimetric plate reader at 490 nm.

The expression of IL-8 was measured by a ligand-specific commercially available ELISA kit that utilizes a quantitative sandwich enzyme immunoassay technique using reagents from R&D systems (Minneapolis, MN).

A 29-plex human cytokine/chemokine (HCYTMAG-60K-PX29) magnetic bead Luminex panel (EMD Millipore, Billerica, MA) were run on cell media (*n* = 3 samples from a representative experiment). All samples were run in duplicate, per the manufacturer’s protocol on the FLEXMAP 3D Luminex platform (Luminex, Austin, TX). Absolute quantification in pg/mL was obtained using a standard curve generated by a five-parameter logistic (5PL) curve fit using xPONENT 4.2 software (Luminex). Fold change values were calculated between treatment groups and the non-treated and NYC EV controls. For fold change calculations, if the cytokines levels in the control group were undetectable, then a minimal detectable level was assigned equal to 0.01 pg/mL. Heatmaps were created using GraphPad.

### Detection of TLR2-dependent NF-kB and IL-8

HEK-hTLR2 cells were plated at 7.5 × 10^4^ cells/well in 96-well plates containing DMEM + 10% heat-inactivated FBS without antibiotics (*n* = 3 samples per condition). The next day, the cells were treated with EVs in DMEM cell culture media for 24 h. For detection of a nuclear factor kappa-B (NF-κB) response (SEAP reporter), cell culture supernatants were incubated with QUANTI-Blue solution (Invivogen) for 1 h, pictures were taken of the plate, and absorbance was read at 630 nm on a SpectraMax i3x plate reader (Molecular Devices). For detection of an IL-8 response (Lucia luciferase reporter), cell culture supernatants were mixed with QUANTI-Luc solution (InvivoGen), and luminescence was read immediately on a SpectraMax i3X plate reader. Additionally, cell death was measured as described above.

### Statistical analysis

All statistical analyses were carried out in GraphPad Prism (GraphPad Software Inc, Version 9.0). A *p*‐value < 0.05 was considered statistically significant. For data that were normally distributed, one-way analysis of variance (ANOVA) was performed. If statistical significance was reached, then pairwise comparison with a Tukey post-hoc test was performed for multiple comparisons. If data were not normally distributed, then the nonparametric Kruskal-Wallis test was used, and multiple comparisons were done using Dunnett’s test.

### Reporting summary

Further information on research design is available in the Nature Research Reporting Summary linked to this article

### Supplementary information

Supplemental Information

Nature Research Reporting Summary

Supplemental Video 1

Supplemental Video 2

Supplemental Video 3

Supplemental Video 4

Supplemental Video 5

Supplemental Video 6

Supplemental Video 7

Supplemental Video 8

Supplemental Video 9

Supplemental Video 10

Supplemental Video 11

Supplemental Video 12

## Data Availability

All data generated or analyzed during this study are included in this published article and its supplementary information files.
