# Supplementary material for: Extracellular vesicles from vaginal Gardnerella vaginalis and Mobiluncus mulieris contain distinct proteomic cargo and induce inflammatory pathways
Source: NPJ Biofilms Microbiomes. 2024 Mar 21;10:28. doi: 10.1038/s41522-024-00502-y (PMC10957959; doi:10.1038/s41522-024-00502-y)
Supplement: Supplementary file 1 — Supplemental Information [file 41522_2024_502_MOESM1_ESM.pdf]

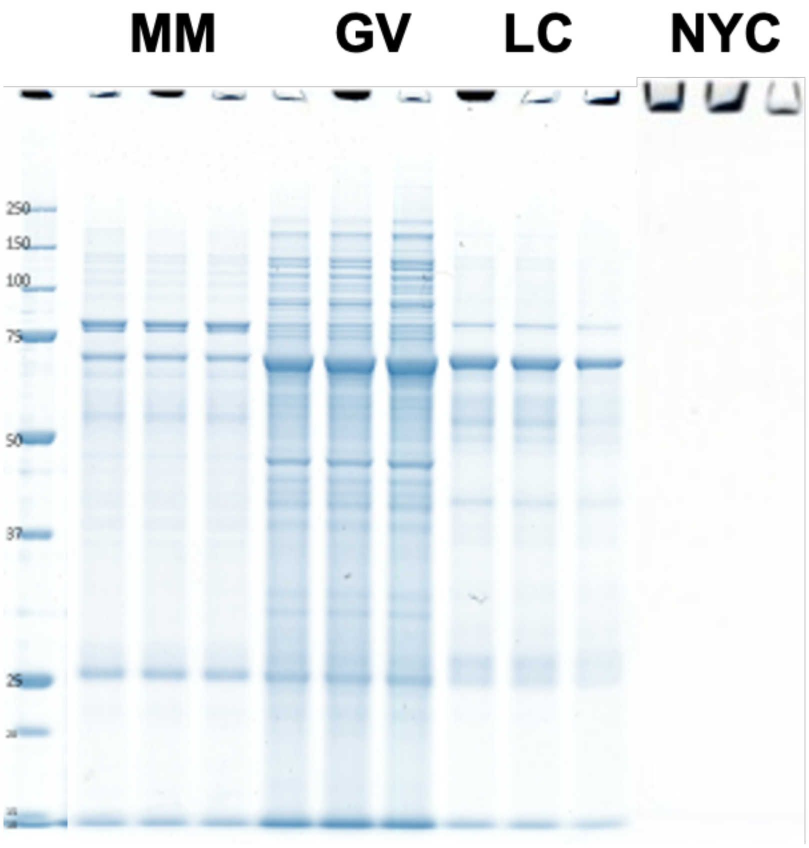

Supplemental Figure 1. Protein profile of bEVs isolated from *M. mulieris* (MM), *G. vaginalis* (GV), *L. crispatus* (LC), and NYC media controls.

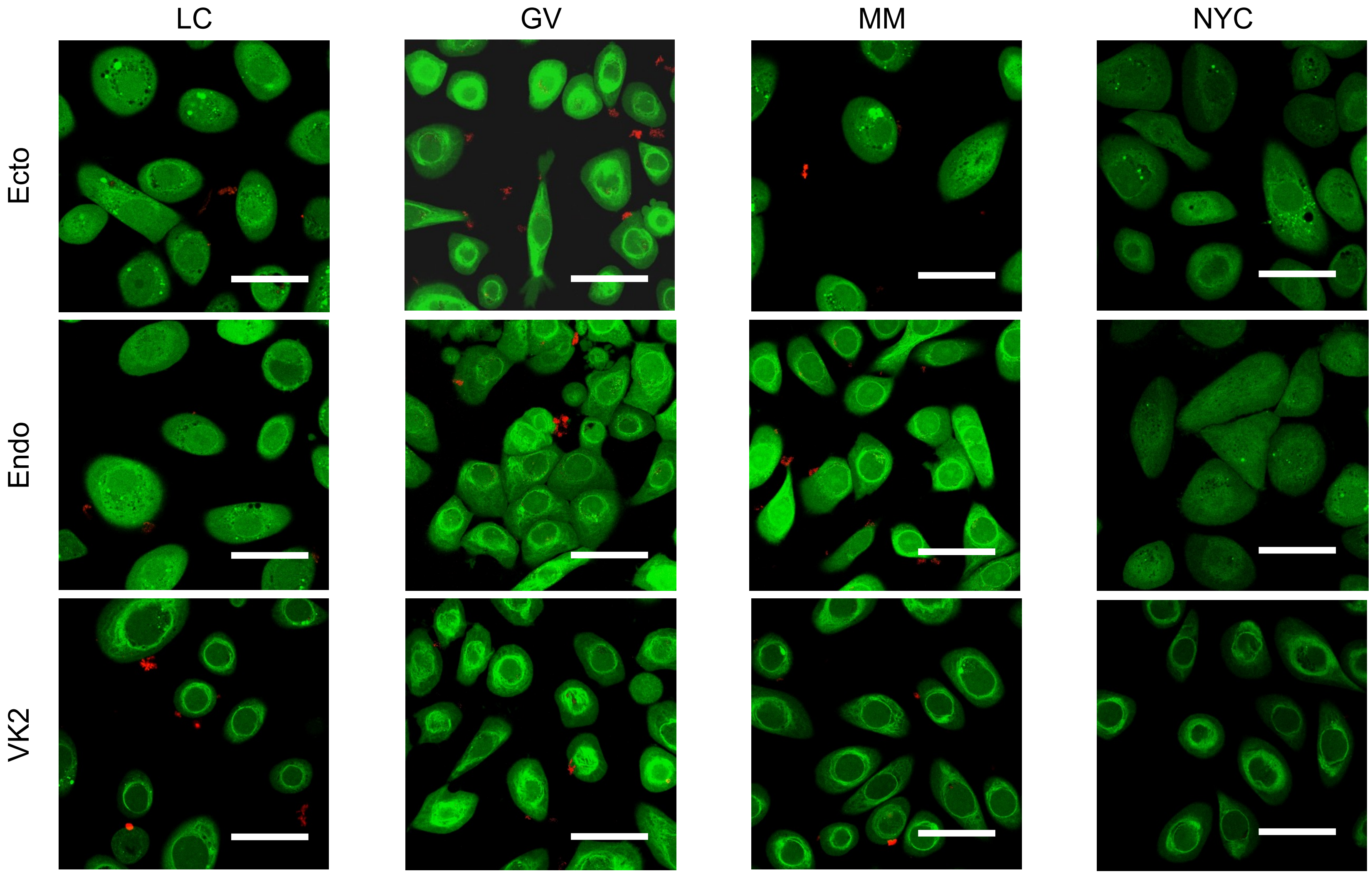

Supplemental Figure 2. Representative images from live videos of bEV (red) uptake by cervicovaginal epithelial cells (green). Video files (.avi), named by cell type and bEV type, are available separately. All scale bars are 20 µm.

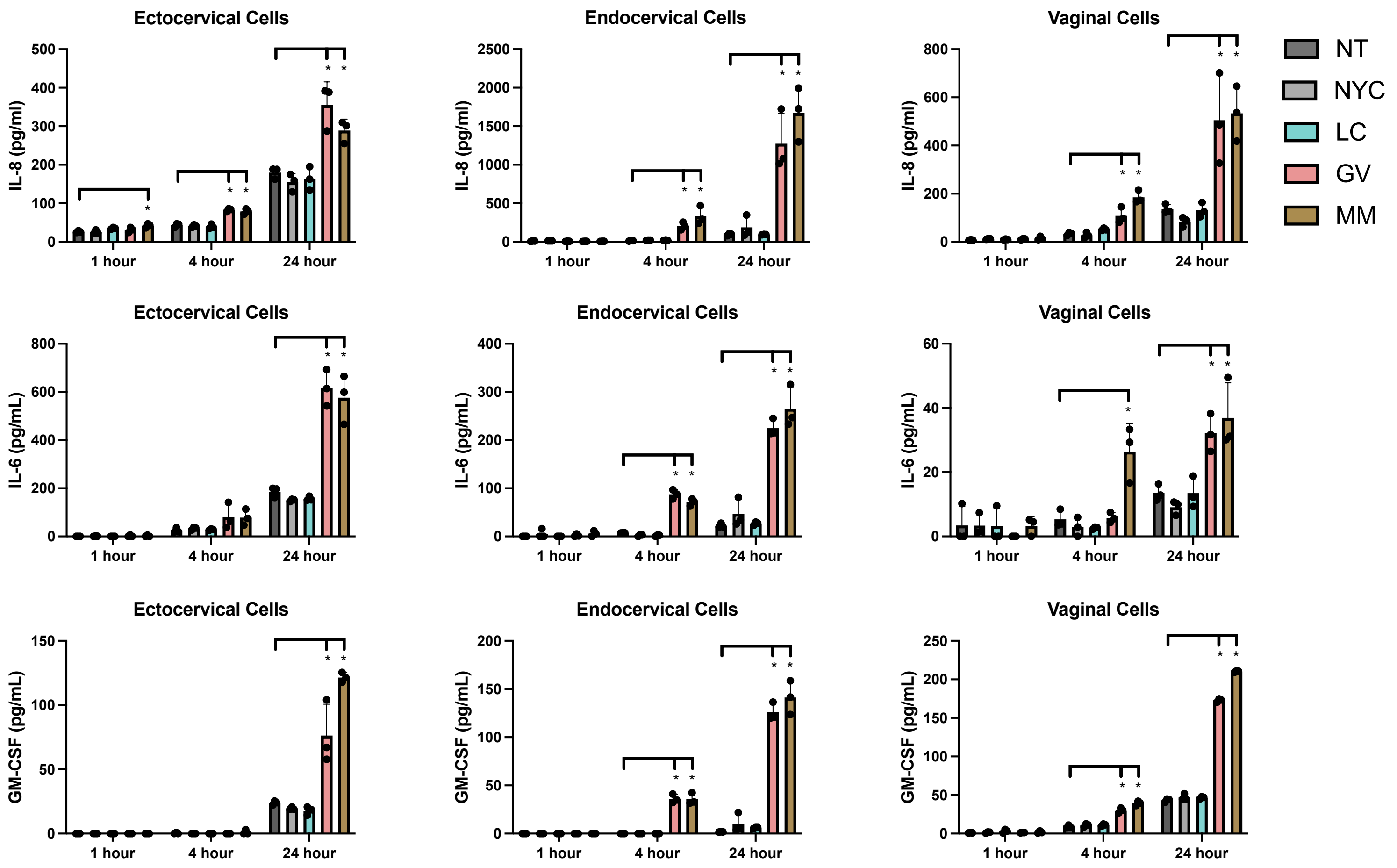

Supplemental Figure 3. Levels of cytokines IL-8, IL-6, and GM-CSF from ectocervical, endocervical, and vaginal epithelial cells after 1, 4, and 24 h of exposure to bEVs. *$p<0.05$ relative to non-treated cells at the same timepoint via one-way ANOVA with Tukey's correction for multiple comparisons.

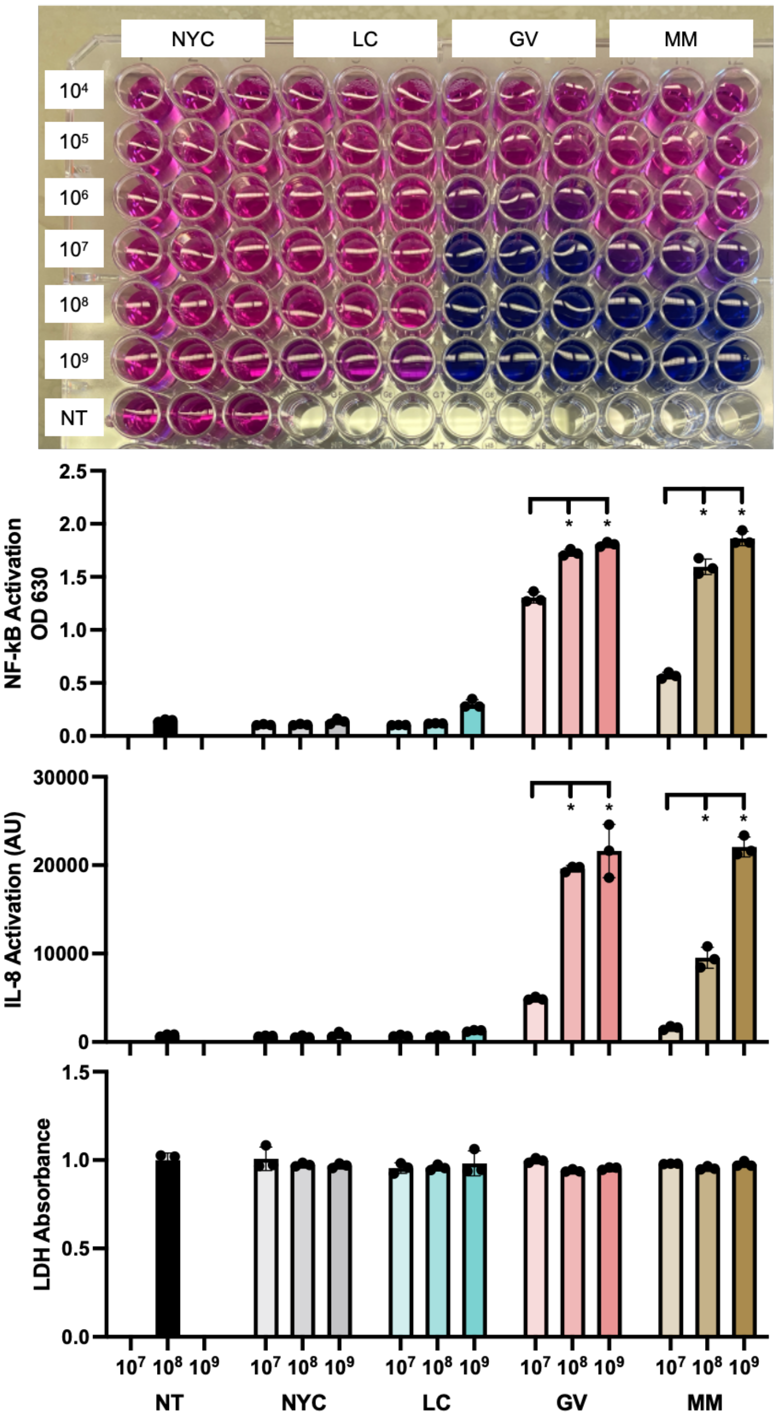

Supplemental Figure 4. 24 h exposure of *G. vaginalis* and *M. mulieris* bEVs to HEK-TLR2 reporter cells resulted in dose-dependent increases in NF-kB activation and IL-8 release without inducing cell death as measured by lactate dehydrogenase (LDH). *$p<0.05$ relative to lowest dose via one-way ANOVA with Tukey's correction for multiple comparisons.

Supplemental Tables

Supplemental Table 1. List of proteins shared between bEVs from *L. crispatus*, *G. vaginalis*, and *M. mulieris*.

**Protein Name**
30S ribosomal protein S13
50S ribosomal protein L3
50S ribosomal protein L4
ABC transporter, ATP-binding protein
ABC transporter, solute-binding protein
ABC transporter, substrate-binding protein
ABC transporter, substrate-binding protein, family 5
adenylate kinase
adenylosuccinate synthase
alanine racemase
alanine--tRNA ligase
aspartate--tRNA ligase
ATP synthase F1, alpha subunit
ATP synthase F1, beta subunit
ATPase family associated with various cellular activities (AAA)
chaperone protein DnaK
chaperonin GroL
cysteine--tRNA ligase
D-ala D-ala ligase N-terminal domain protein
DNA polymerase III, beta subunit
DNA-directed RNA polymerase, alpha subunit
DNA-directed RNA polymerase, beta subunit
DNA-directed RNA polymerase, beta' subunit
dTMP kinase
fructose-bisphosphate aldolase, class-II
glutamate--tRNA ligase
glutamine synthetase, type I
glutamine-fructose-6-phosphate transaminase (isomerizing)
glyceraldehyde-3-phosphate dehydrogenase, type I
GMP synthase (glutamine-hydrolyzing) domain protein
GTP-binding protein TypA
GTP-binding protein YchF
histidine triad domain protein
histidine--tRNA ligase
homoserine dehydrogenase
hypothetical protein
hypoxanthine phosphoribosyltransferase
isoleucine--tRNA ligase
L-lactate dehydrogenase
leucine--tRNA ligase
lysine--tRNA ligase
methionine adenosyltransferase
methionine aminopeptidase, type I
methionine--tRNA ligase
methionyl-tRNA formyltransferase
nitroreductase family protein

NlpC/P60 family protein
oxidoreductase, aldo/keto reductase family protein
peptide deformylase
phenylalanine--tRNA ligase, beta subunit
phosphate acetyltransferase
phosphoenolpyruvate carboxykinase (GTP)
phosphoglucosamine mutase
phosphoglycerate kinase
phosphoglycerate mutase 1 family
phosphopyruvate hydratase
proline--tRNA ligase
pyruvate kinase
ribonucleoside-diphosphate reductase, alpha subunit
ribonucleoside-diphosphate reductase, beta subunit
ribose-phosphate diphosphokinase
ribosomal protein L1
ribosomal protein L10
ribosomal protein L13
ribosomal protein L14
ribosomal protein L20
ribosomal protein L5
ribosomal protein L7/L12
ribosomal protein S10
ribosomal protein S2
ribosomal protein S3
ribosomal protein S5
ribosomal subunit interface protein
RNA polymerase sigma factor RpoD
serine--tRNA ligase
signal recognition particle protein
Sua5/YciO/YrdC/YwlC family protein
threonine synthase
threonine--tRNA ligase
transcription termination factor NusA
translation elongation factor G
translation elongation factor Tu
trigger factor
tryptophan--tRNA ligase
tyrosine--tRNA ligase
UDP-glucose 4-epimerase
UDP-N-acetylmuramoyl-L-alanine--D-glutamate ligase
UDP-N-acetylmuramoyl-tripeptide--D-alanyl-D- alanine ligase
UMP kinase
uracil phosphoribosyltransferase

Supplemental Table 2. List of proteins, their abundance, and their associated functional pathway identified in *G. vaginalis* bEVs.

Protein Name	Abundance (ppm)	Functional pathway
[glutamate--ammonia-ligase] adenylyltransferase	2556	Coenzyme transport & metabolism
1,4-alpha-glucan branching enzyme	6893	Carbohydrate transport & metabolism
16S rRNA methyltransferase GidB	852	Translation
16S rRNA processing protein RimM	232	Translation
2-dehydropantoate 2-reductase	697	Coenzyme transport & metabolism
2-dehydropantoate 2-reductase	2014	Coenzyme transport & metabolism
3-dehydroquinate dehydratase, type II	155	Amino acid transport & metabolism
3-dehydroquinate synthase	1162	Coenzyme transport & metabolism
3-deoxy-7-phosphoheptulonate synthase	1162	Amino acid transport & metabolism
3-phosphoshikimate 1-carboxyvinyltransferase	774	Amino acid transport & metabolism
30S ribosomal protein S1	13166	Translation
30S ribosomal protein S13	310	Translation
30S ribosomal protein S17	155	Translation
4-alpha-glucanotransferase	620	Carbohydrate transport & metabolism
50S ribosomal protein L3	697	Translation
50S ribosomal protein L4	1084	Translation
6-phosphogluconolactonase	465	Carbohydrate transport & metabolism
ABC transporter, ATP-binding protein	620	Cell wall/ membrane
ABC transporter, ATP-binding protein	620	Carbohydrate transport & metabolism
ABC transporter, ATP-binding protein	697	Defense mechanisms
ABC transporter, ATP-binding protein	1769	Inorganic ion transport & metabolism
ABC transporter, ATP-binding protein	7048	Amino acid transport & metabolism
ABC transporter, permease protein	155	Amino acid transport & metabolism
ABC transporter, solute-binding protein	3485	Carbohydrate transport & metabolism

ABC transporter, substrate-binding protein	852	Inorganic ion transport & metabolism
ABC transporter, substrate-binding protein, family 3	1007	Signal transduction mechanisms
ABC transporter, substrate-binding protein, family 3	1007	Amino acid transport & metabolism
ABC transporter, substrate-binding protein, family 5	3717	Amino acid transport & metabolism
acetate kinase	10068	Coenzyme transport & metabolism
acetyl-CoA C-acetyltransferase	852	Lipid transport & metabolism
acetyltransferase, GNAT family	151	Translation
acetyltransferase, GNAT family	155	Transcription
acetyltransferase, GNAT family	308	Replication, recombination, and repair
Acyltransferase	387	Lipid transport & metabolism
adenine phosphoribosyltransferase	929	Nucleotide transport & metabolism
adenylate kinase	155	Nucleotide transport & metabolism
adenylosuccinate synthase	2866	Nucleotide transport & metabolism
alanine racemase	929	Cell wall/ membrane
alanine--tRNA ligase	9603	Translation
alcohol dehydrogenase, iron-dependent	1007	Energy production & conversion
aldehyde dehydrogenase (NAD) family protein	7048	Energy production & conversion
aldose 1-epimerase	852	Carbohydrate transport & metabolism
alkyl hydroperoxide reductase, F subunit	154	Posttranslational modification
alkyl hydroperoxide reductase, F subunit	1549	Posttranslational modification
alpha amylase, catalytic domain protein	465	Carbohydrate transport & metabolism
alpha amylase, catalytic domain protein	1162	Cell wall/ membrane
amidohydrolase family protein	1084	Nucleotide transport & metabolism
amidophosphoribosyltransferase	1239	Nucleotide transport & metabolism
amino acid kinase family	465	Amino acid transport & metabolism
amino acid permease	232	Amino acid transport & metabolism
aminoacyl-tRNA hydrolase	774	Translation
aminopeptidase P domain protein	1239	Amino acid transport & metabolism

aminotransferase, class I/II	2788	Amino acid transport & metabolism
AMP-binding enzyme	3872	Lipid transport & metabolism
anaerobic ribonucleoside-triphosphate reductase	5576	Nucleotide transport & metabolism
anaerobic ribonucleoside-triphosphate reductase activating protein	620	Posttranslational modification
anchored repeat ABC transporter, substrate-binding protein	154	Inorganic ion transport & metabolism
arginine--tRNA ligase	1317	Translation
argininosuccinate lyase	929	Amino acid transport & metabolism
argininosuccinate synthase	1084	Amino acid transport & metabolism
arylsulfatase	151	Cell wall/ membrane
aspartate carbamoyltransferase	1859	Nucleotide transport & metabolism
aspartate--tRNA ligase	7357	Translation
aspartate-semialdehyde dehydrogenase	2246	Amino acid transport & metabolism
aspartyl/glutamyl-tRNA(Asn/Gln) amidotransferase, A subunit	3485	Nucleotide transport & metabolism
aspartyl/glutamyl-tRNA(Asn/Gln) amidotransferase, B subunit	5034	Translation
ATP synthase F1, alpha subunit	542	Energy production & conversion
ATP synthase F1, alpha subunit	3408	Energy production & conversion
ATP synthase F1, beta subunit	852	Energy production & conversion
ATP synthase F1, beta subunit	5421	Energy production & conversion
ATP synthase F1, delta subunit	620	Energy production & conversion
ATP synthase F1, gamma subunit	1084	Energy production & conversion
ATP-dependent chaperone protein ClpB	232	Posttranslational modification
ATP-dependent metallopeptidase HflB	1007	Posttranslational modification
ATPase family associated with various cellular activities (AAA)	226	Posttranslational modification
ATPase family associated with various cellular activities (AAA)	6041	Posttranslational modification
ATPase/histidine kinase/DNA gyrase B/HSP90 domain protein	774	Signal transduction mechanisms
Beta galactosidase small chain	774	Carbohydrate transport & metabolism
bifunctional protein FolC	1007	Coenzyme transport & metabolism
branched-chain amino acid transport system II carrier protein	154	Intracellular trafficking

branched-chain-amino-acid transaminase	2169	Amino acid transport & metabolism
BRCA1 C-terminal domain protein	620	Replication, recombination, and repair
Carbamoyl-phosphate synthase L chain, ATP binding domain protein	2711	Lipid transport & metabolism
carbamoyl-phosphate synthase, large subunit	154	Nucleotide transport & metabolism
carbamoyl-phosphate synthase, large subunit	154	Amino acid transport & metabolism
carbamoyl-phosphate synthase, large subunit	4492	Nucleotide transport & metabolism
carbamoyl-phosphate synthase, large subunit	4492	Amino acid transport & metabolism
carbamoyl-phosphate synthase, small subunit	1084	Nucleotide transport & metabolism
carbonate dehydratase	620	Inorganic ion transport & metabolism
carboxyl transferase domain protein	310	Lipid transport & metabolism
cell division ATP-binding protein FtsE	1549	Cell division
cell division protein FtsZ	3020	Cell division
cell envelope-like function transcriptional attenuator common domain protein	387	Transcription
CHAP domain protein	1239	Cell division
chaperone protein DnaK	154	Posttranslational modification
chaperonin GroL	1162	Posttranslational modification
chaperonin GroS	154	Posttranslational modification
chorismate synthase	155	Amino acid transport & metabolism
chromosomal replication initiator protein DnaA	1704	Replication, recombination, and repair
Cna protein B-type domain protein	697	Cell wall/ membrane
co-chaperone GrpE	542	Posttranslational modification
cobalt ABC transporter, ATP-binding protein	697	Inorganic ion transport & metabolism
CobQ/CobB/MinD/ParA nucleotide binding domain protein	852	Cell division
cold-shock DNA-binding domain protein	151	Transcription
conserved hypothetical protein, YfiH family	697	Secondary metabolites biosynthesis, transport and catabolism
coproporphyrinogen dehydrogenase	310	Coenzyme transport & metabolism
CRISPR system CASCADE complex protein CasC	852	Replication, recombination, and repair

crossover junction endodeoxyribonuclease RuvC	465	Replication, recombination, and repair
CTP synthase	5034	Nucleotide transport & metabolism
cyclomaltodextrinase	1471	Carbohydrate transport & metabolism
cysteine desulfurase, SufS subfamily	1704	Amino acid transport & metabolism
cysteine--tRNA ligase	2091	Translation
D-ala D-ala ligase N-terminal domain protein	1936	Nucleotide transport & metabolism
D-alanyl-D-alanine carboxypeptidase/D-alanyl-D-alanine-endopeptidase	697	Cell wall/ membrane
D-xylulose 5-phosphate/D-fructose 6-phosphate phosphoketolase	16264	Carbohydrate transport & metabolism
dCTP deaminase	387	Nucleotide transport & metabolism
DEAD/DEAH box helicase	310	Replication, recombination, and repair
DEAD/DEAH box helicase	697	Transcription
DEAD/DEAH box helicase	697	Translation
di-trans,poly-cis-decaprenylcistransferase	465	Coenzyme transport & metabolism
dihydrodipicolinate synthetase family	542	Cell wall/ membrane
dihydrodipicolinate synthetase family	542	Amino acid transport & metabolism
dihydrofolate reductase	697	Coenzyme transport & metabolism
dihydroorotate oxidase	929	Nucleotide transport & metabolism
dimethyladenosine transferase	1007	Translation
dipeptidase	4569	Amino acid transport & metabolism
diphosphomevalonate decarboxylase	774	Lipid transport & metabolism
divergent AAA domain protein	155	Transcription
DivIVA domain protein	232	Defense mechanisms
DivIVA domain repeat protein	1162	Intracellular trafficking
DivIVA domain repeat protein	1162	Cell motility
DNA (cytosine-5-)-methyltransferase	542	Replication, recombination, and repair
DNA gyrase, A subunit	2091	Replication, recombination, and repair
DNA gyrase, B subunit	2866	Replication, recombination, and repair
DNA gyrase, B subunit, C-terminal domain protein	620	Replication, recombination, and repair

DNA gyrase/topoisomerase IV, A subunit	155	Replication, recombination, and repair
DNA ligase (NAD+)	2633	Replication, recombination, and repair
DNA polymerase III, alpha subunit	2943	Replication, recombination, and repair
DNA polymerase III, beta subunit	3795	Replication, recombination, and repair
DNA polymerase III, delta' subunit	620	Replication, recombination, and repair
DNA polymerase III, subunit gamma and tau	1471	Replication, recombination, and repair
DNA primase	1704	Replication, recombination, and repair
DNA repair protein RadA	310	Posttranslational modification
DNA replication and repair protein RecF	232	Replication, recombination, and repair
DNA-binding helix-turn-helix protein	232	Transcription
DNA-binding protein HB1	232	Replication, recombination, and repair
DNA-binding regulatory protein, YebC/PmpR family	387	Transcription
DNA-directed DNA polymerase	1317	Replication, recombination, and repair
DNA-directed RNA polymerase, alpha subunit	387	Transcription
DNA-directed RNA polymerase, alpha subunit	4105	Transcription
DNA-directed RNA polymerase, beta subunit	852	Transcription
DNA-directed RNA polymerase, beta subunit	11462	Transcription
DNA-directed RNA polymerase, beta' subunit	697	Transcription
DNA-directed RNA polymerase, beta' subunit	697	Transcription
DNA-directed RNA polymerase, beta' subunit	13786	Transcription
DNA-directed RNA polymerase, omega subunit	232	Transcription
dTDP-glucose 4,6-dehydratase	4182	Cell wall/ membrane
dTMP kinase	465	Nucleotide transport & metabolism
endopeptidase Clp	310	Posttranslational modification
excinuclease ABC, B subunit	542	Replication, recombination, and repair
exodeoxyribonuclease VII, large subunit	231	Replication, recombination, and repair
exonuclease	155	Replication, recombination, and repair
Exonuclease VII, large subunit	155	Replication, recombination, and repair

FAD binding domain protein	154	Energy production & conversion
FAD dependent oxidoreductase	4105	Coenzyme transport & metabolism
FAH family protein	387	Secondary metabolites biosynthesis, transport and catabolism
FCD domain protein	387	Transcription
FemAB family protein	387	Defense mechanisms
FeS assembly ATPase SufC	2633	Posttranslational modification
FeS assembly protein SufB	2246	Posttranslational modification
FeS assembly protein SufD	1549	Posttranslational modification
FHA domain protein	929	Signal transduction mechanisms
formate C-acetyltransferase	387	Energy production & conversion
formate C-acetyltransferase	9061	Energy production & conversion
fructosamine kinase	1549	Carbohydrate transport & metabolism
fructose-bisphosphate aldolase, class II	1007	Carbohydrate transport & metabolism
FtsK/SpoIIIE family protein	387	Cell division
galactokinase	1704	Carbohydrate transport & metabolism
glucosamine-6-phosphate deaminase	1007	Carbohydrate transport & metabolism
glucose-1-phosphate thymidylyltransferase	1859	Coenzyme transport & metabolism
glucose-6-phosphate dehydrogenase	2401	Carbohydrate transport & metabolism
glucose-6-phosphate isomerase	387	Carbohydrate transport & metabolism
glucose-6-phosphate isomerase	3175	Carbohydrate transport & metabolism
glutamate racemase	1394	Cell wall/ membrane
glutamate--tRNA ligase	4724	Translation
glutamate-5-semialdehyde dehydrogenase	1859	Amino acid transport & metabolism
glutamine synthetase, type I	697	Amino acid transport & metabolism
glutamine-fructose-6-phosphate transaminase (isomerizing)	3330	Cell wall/ membrane
glyceraldehyde-3-phosphate dehydrogenase, type I	6815	Carbohydrate transport & metabolism
glycerol-3-phosphate dehydrogenase [NAD(P)+]	1471	Lipid transport & metabolism

glycine oxidase ThiO	232	Amino acid transport & metabolism
glycine--tRNA ligase	2711	Translation
glycogen debranching enzyme GlgX	2478	Carbohydrate transport & metabolism
glycosyl hydrolase family 20, catalytic domain protein	542	Carbohydrate transport & metabolism
glycosyl hydrolase family 25	852	Cell wall/ membrane
glycosyl hydrolase, family 31	1007	Carbohydrate transport & metabolism
glycosyltransferase, group 1 family protein	310	Cell wall/ membrane
glycosyltransferase, group 2 family protein	465	Cell wall/ membrane
GMP synthase (glutamine-hydrolyzing) domain protein	226	Nucleotide transport & metabolism
GMP synthase (glutamine-hydrolyzing) domain protein	2866	Nucleotide transport & metabolism
Gram-positive signal peptide protein, YSIRK family	310	Cell division
GTP-binding protein LepA	3330	Cell wall/ membrane
GTP-binding protein TypA	697	Signal transduction mechanisms
GTP-binding protein TypA	6351	Signal transduction mechanisms
GTP-binding protein YchF	542	Translation
GTP-binding protein YchF	2401	Translation
guanosine pentaphosphate synthetase I/polyribonucleotide nucleotidyltransferase	542	Translation
guanosine pentaphosphate synthetase I/polyribonucleotide nucleotidyltransferase	9294	Translation
guanylate kinase	155	Nucleotide transport & metabolism
HAD hydrolase, family IIA	1239	Carbohydrate transport & metabolism
Ham1 family protein	929	Nucleotide transport & metabolism
histidine triad domain protein	387	Carbohydrate transport & metabolism
histidine triad domain protein	387	Nucleotide transport & metabolism
histidine--tRNA ligase	3872	Translation
Holliday junction DNA helicase RuvA	155	Replication, recombination, and repair
Holliday junction DNA helicase RuvB	852	Replication, recombination, and repair
homoserine dehydrogenase	1549	Amino acid transport & metabolism
homoserine kinase	308	Amino acid transport & metabolism

HTH domain protein	465	Carbohydrate transport & metabolism
hydrolase, NUDIX family	232	Nucleotide transport & metabolism
hydrolase, NUDIX family	387	Replication, recombination, and repair
hydroxymethylglutaryl-CoA reductase, degradative	310	Lipid transport & metabolism
hydroxymethylglutaryl-CoA synthase	1239	Lipid transport & metabolism
hypothetical protein	231	Signal transduction mechanisms
hypothetical protein	387	Posttranslational modification
hypothetical protein	620	Carbohydrate transport & metabolism
hypothetical protein	929	Secondary metabolites biosynthesis, transport and catabolism
hypothetical protein	980	Cell division
hypothetical protein	1007	Cell wall/ membrane
hypothetical protein	1084	Translation
hypothetical protein	1162	Inorganic ion transport & metabolism
hypothetical protein	2091	Transcription
hypothetical protein	3175	Replication, recombination, and repair
hypothetical protein	6893	Lipid transport & metabolism
hypoxanthine phosphoribosyltransferase	774	Nucleotide transport & metabolism
IMP dehydrogenase family protein	465	Nucleotide transport & metabolism
inorganic diphosphatase	852	Energy production & conversion
inosine-5'-monophosphate dehydrogenase	155	Nucleotide transport & metabolism
inosine-5'-monophosphate dehydrogenase	3330	Nucleotide transport & metabolism
Inosine-uridine preferring nucleoside hydrolase	929	Nucleotide transport & metabolism
iojap-like protein	310	Translation
isoleucine--tRNA ligase	5034	Translation
isopentenyl-diphosphate delta-isomerase, type 2	1471	Energy production & conversion
kinase domain protein	542	Signal transduction mechanisms
kinase domain protein	542	Replication, recombination, and repair

kinase domain protein	542	Transcription
kinase, PfkB family	2091	Carbohydrate transport & metabolism
kojibiose phosphorylase	387	Carbohydrate transport & metabolism
L-arabinose isomerase	1317	Carbohydrate transport & metabolism
L-lactate dehydrogenase	3253	Energy production & conversion
L-serine ammonia-lyase	1626	Amino acid transport & metabolism
lactate/malate dehydrogenase, NAD binding domain protein	3253	Energy production & conversion
leucine--tRNA ligase	8906	Translation
low molecular weight phosphotyrosine protein phosphatase	697	Signal transduction mechanisms
LPXTG-motif cell wall anchor domain protein	155	Cell wall/ membrane
lysine--tRNA ligase	310	Translation
lysine--tRNA ligase	9449	Translation
LysR substrate binding domain protein	542	Transcription
MaoC-like protein	10533	Lipid transport & metabolism
membrane alanyl aminopeptidase	387	Amino acid transport & metabolism
methionine adenosyltransferase	1704	Coenzyme transport & metabolism
methionine aminopeptidase, type I	1239	Amino acid transport & metabolism
methionine--tRNA ligase	3872	Translation
methionyl-tRNA formyltransferase	697	Translation
methyltransferase domain protein	852	Translation
methyltransferase small domain protein	154	Translation
Mur ligase middle domain protein	774	Cell wall/ membrane
N-acetylglucosamine-6-phosphate deacetylase	1317	Carbohydrate transport & metabolism
N-acylglucosamine 2-epimerase	387	Carbohydrate transport & metabolism
NAD dependent epimerase/dehydratase family protein	1704	Cell wall/ membrane
NAD dependent epimerase/dehydratase family protein	1704	Carbohydrate transport & metabolism
NAD(+)/NADH kinase	387	Coenzyme transport & metabolism
NAD+ synthase	852	Coenzyme transport & metabolism

NADH oxidase	4492	Energy production & conversion
nicotinate phosphoribosyltransferase	310	Nucleotide transport & metabolism
nitroreductase family protein	542	Energy production & conversion
NLPA lipoprotein	620	Inorganic ion transport & metabolism
NlpC/P60 family protein	1007	Cell wall/ membrane
NMT1/THI5-like protein	1704	Inorganic ion transport & metabolism
Obg family GTPase CgtA	151	Transcription
orotate phosphoribosyltransferase	1471	Nucleotide transport & metabolism
orotidine 5'-phosphate decarboxylase	774	Nucleotide transport & metabolism
oxidoreductase NAD-binding domain protein	929	Energy production & conversion
oxidoreductase, aldo/keto reductase family protein	1549	Energy production & conversion
oxidoreductase, short chain dehydrogenase/reductase family protein	620	Secondary metabolites biosynthesis, transport and catabolism
oxidoreductase, short chain dehydrogenase/reductase family protein	620	Lipid transport & metabolism
pantothenate kinase	310	Coenzyme transport & metabolism
ParB-like protein	387	Transcription
penicillin-binding protein A	232	Cell wall/ membrane
penicillin-binding protein dimerization domain protein	465	Cell wall/ membrane
penicillin-binding protein, transpeptidase domain protein	1084	Cell wall/ membrane
peptidase C1-like family	1239	Amino acid transport & metabolism
peptidase dimerization domain protein	155	Amino acid transport & metabolism
peptidase family M13	852	Posttranslational modification
peptide chain release factor 1	226	Translation
peptide chain release factor 2	542	Translation
peptide deformylase	542	Translation
peptidyl-prolyl cis-trans isomerase, cyclophilin-type	697	Carbohydrate transport & metabolism
peptidyl-prolyl cis-trans isomerase, FKBP-type	1239	Cell wall/ membrane
phenylalanine--tRNA ligase, alpha subunit	542	Translation

phenylalanine--tRNA ligase, beta subunit	3175	Translation
phenylalanyl-tRNA synthetase, beta subunit	310	Translation
PhoH family protein	310	Signal transduction mechanisms
phosphate ABC transporter, ATP-binding protein	697	Inorganic ion transport & metabolism
phosphate ABC transporter, phosphate-binding protein PstS	542	Inorganic ion transport & metabolism
phosphate acetyltransferase	465	Energy production & conversion
phosphate transport system regulatory protein PhoU	1549	Inorganic ion transport & metabolism
phosphoenolpyruvate carboxykinase (GTP)	4337	Coenzyme transport & metabolism
phosphoglucomutase, alpha-D-glucose phosphate-specific	9681	Carbohydrate transport & metabolism
phosphogluconate dehydrogenase (decarboxylating)	852	Coenzyme transport & metabolism
phosphogluconate dehydrogenase (decarboxylating)	1549	Carbohydrate transport & metabolism
phosphoglucosamine mutase	3408	Carbohydrate transport & metabolism
phosphoglycerate kinase	774	Nucleotide transport & metabolism
phosphoglycerate mutase 1 family	5189	Carbohydrate transport & metabolism
phosphoglycerate mutase family protein	1007	Carbohydrate transport & metabolism
phosphomethylpyrimidine kinase	155	Transcription
phosphomethylpyrimidine kinase	155	Coenzyme transport & metabolism
phosphopyruvate hydratase	8597	Carbohydrate transport & metabolism
phosphoribosylamine--glycine ligase	542	Nucleotide transport & metabolism
phosphoribosylaminoimidazole carboxylase, ATPase subunit	2169	Nucleotide transport & metabolism
phosphoribosylaminoimidazolesuccinocarboxamide synthase	1626	Nucleotide transport & metabolism
phosphoribosylformylglycinamidine cyclo-ligase	1394	Nucleotide transport & metabolism
phosphoribosylformylglycinamidine synthase	5809	Nucleotide transport & metabolism
phosphorylase, glycogen/starch/alpha-glucan family	11152	Carbohydrate transport & metabolism
phosphorylase, Pnp/Udp family	542	Nucleotide transport & metabolism
polyphosphate kinase 1	4492	Inorganic ion transport & metabolism
polyprenyl synthetase	697	Coenzyme transport & metabolism
POTRA domain protein, FtsQ-type	155	Cell division

Ppx/GppA phosphatase family protein	2556	Inorganic ion transport & metabolism
Ppx/GppA phosphatase family protein	2556	Nucleotide transport & metabolism
prephenate dehydratase	232	Amino acid transport & metabolism
prephenate dehydrogenase	232	Amino acid transport & metabolism
preprotein translocase, SecA subunit	6738	Intracellular trafficking
preprotein translocase, YajC subunit	231	Intracellular trafficking
proline--tRNA ligase	774	Translation
prolipoprotein diacylglyceryl transferase	232	Cell wall/ membrane
protein phosphatase 2C	232	Signal transduction mechanisms
protein-(glutamine-N5) methyltransferase, release factor-specific	465	Translation
pseudouridine synthase, RluA family	232	Translation
pseudouridylate synthase	232	Translation
pullulanase, type I	852	Carbohydrate transport & metabolism
putative 5-methyltetrahydropteroyltriglutamate--homocysteine S-methyltransferase	310	Amino acid transport & metabolism
putative 6-phospho 3-hexuloisomerase	154	Cell wall/ membrane
putative adenylosuccinate lyase	1626	Nucleotide transport & metabolism
putative ATP-dependent DNA helicase PcrA	852	Replication, recombination, and repair
putative ATP-dependent DNA helicase RecG	929	Replication, recombination, and repair
putative chaperone protein DnaJ	1162	Posttranslational modification
putative CRISPR system CASCADE complex protein CasA	1162	Replication, recombination, and repair
putative dGTPase	387	Nucleotide transport & metabolism
putative dihydrodipicolinate synthase	542	Cell wall/ membrane
putative dihydrodipicolinate synthase	542	Amino acid transport & metabolism
putative dihydroorotate oxidase	774	Nucleotide transport & metabolism
putative DNA polymerase III, delta subunit	465	Replication, recombination, and repair
putative DNA repair protein RecN	774	Replication, recombination, and repair
putative dTDP-4-dehydrorhamnose reductase	1317	Cell wall/ membrane
putative eukaryotic phosphomannomutase	542	Signal transduction mechanisms

putative exodeoxyribonuclease III	1084	Replication, recombination, and repair
putative formate--tetrahydrofolate ligase	6893	Nucleotide transport & metabolism
putative glutamyl-queuosine tRNA(Asp) synthetase	1007	Translation
putative glycoprotease GCP	1162	Posttranslational modification
putative histidinol-phosphate transaminase	1162	Amino acid transport & metabolism
putative inositol 1-phosphate synthase	1626	Lipid transport & metabolism
putative opcA protein	1317	Carbohydrate transport & metabolism
putative phosphonate C-P lyase system protein PhnK	697	Amino acid transport & metabolism
putative phosphonate C-P lyase system protein PhnK	2401	Inorganic ion transport & metabolism
putative phosphoribosylaminoimidazolecarboxamide formyltransferase/IMP cyclohydrolase	620	Nucleotide transport & metabolism
putative phosphoribosylglycinamide formyltransferase 2	1007	Nucleotide transport & metabolism
putative pyroglutamyl-peptidase I	542	Posttranslational modification
putative ribokinase	852	Coenzyme transport & metabolism
putative RNA methyltransferase, TrmH family, group 2	1394	Translation
putative serine O-acetyltransferase	310	Amino acid transport & metabolism
putative undecaprenyldiphospho-muramoylpentapeptide beta-N-acetylglucosaminyltransferase	387	Cell wall/ membrane
putative YjeF domain protein	620	Coenzyme transport & metabolism
pyridine nucleotide-disulfide oxidoreductase	1859	Energy production & conversion
pyridoxal 5'-phosphate synthase, glutaminase subunit Pdx2	1626	Coenzyme transport & metabolism
pyridoxal 5'-phosphate synthase, synthase subunit Pdx1	465	Coenzyme transport & metabolism
pyrroline-5-carboxylate reductase	465	Amino acid transport & metabolism
pyruvate formate-lyase 1-activating enzyme	1162	Energy production & conversion
pyruvate kinase	17193	Carbohydrate transport & metabolism
RecA protein	2711	Replication, recombination, and repair
recombination factor protein RarA	774	Replication, recombination, and repair
recombination protein RecR	310	Replication, recombination, and repair
RelA/SpoT family protein	2246	Signal transduction mechanisms

RelA/SpoT family protein	2246	Transcription
repeat protein	2943	Intracellular trafficking
repeat protein	2943	Cell motility
replicative DNA helicase	1084	Replication, recombination, and repair
repressor LexA	232	Transcription
response regulator receiver domain protein	155	Transcription
response regulator receiver domain protein	774	Signal transduction mechanisms
rhamnan synthesis protein F	1162	Cell wall/ membrane
riboflavin kinase	1394	Coenzyme transport & metabolism
ribonuclease HII	465	Replication, recombination, and repair
ribonuclease III	155	Translation
ribonuclease, Rne/Rng family	2556	Translation
ribonucleoside-diphosphate reductase, alpha subunit	232	Nucleotide transport & metabolism
ribonucleoside-diphosphate reductase, alpha subunit	3175	Nucleotide transport & metabolism
ribonucleoside-diphosphate reductase, beta subunit	3717	Nucleotide transport & metabolism
ribose 5-phosphate isomerase A	2788	Carbohydrate transport & metabolism
ribose-phosphate diphosphokinase	1471	Nucleotide transport & metabolism
ribosomal protein L1	2091	Translation
ribosomal protein L10	1471	Translation
ribosomal protein L11	465	Translation
ribosomal protein L13	620	Translation
ribosomal protein L14	387	Translation
ribosomal protein L15	310	Translation
ribosomal protein L16	697	Translation
ribosomal protein L17	310	Translation
ribosomal protein L18	231	Translation
ribosomal protein L19	620	Translation
ribosomal protein L2	310	Translation

ribosomal protein L20	232	Translation
ribosomal protein L21	310	Translation
ribosomal protein L22	310	Translation
ribosomal protein L23	310	Translation
ribosomal protein L24	232	Translation
ribosomal protein L25, Ctc-form	1239	Translation
ribosomal protein L28	155	Translation
ribosomal protein L31	231	Translation
ribosomal protein L5	1704	Translation
ribosomal protein L6	620	Translation
ribosomal protein L7/L12	697	Translation
ribosomal protein L9	465	Translation
ribosomal protein S10	852	Translation
ribosomal protein S15	310	Translation
ribosomal protein S16	465	Translation
ribosomal protein S2	3485	Translation
ribosomal protein S3	852	Translation
ribosomal protein S4	929	Translation
ribosomal protein S5	2169	Translation
ribosomal protein S6	387	Translation
ribosomal protein S7	620	Translation
ribosomal protein S8	1317	Translation
ribosomal protein S9	155	Translation
ribosomal RNA large subunit methyltransferase J	155	Translation
ribosomal subunit interface protein	620	Translation
ribosomal-protein-alanine acetyltransferase	155	Transcription
ribosome biogenesis GTPase Der	3253	Nucleotide transport & metabolism
ribulose-phosphate 3-epimerase	232	Carbohydrate transport & metabolism

RNA methyltransferase	774	Translation
RNA methyltransferase, TrmH family	929	Translation
RNA methyltransferase, TrmH family, group 3	1239	Translation
RNA polymerase sigma factor RpoD	1007	Transcription
RNA polymerase sigma factor RpoD	3020	Transcription
ROK family protein	3330	Transcription
ROK family protein	3330	Carbohydrate transport & metabolism
S-adenosyl-L-homocysteine hydrolase, NAD binding domain protein	1781	Coenzyme transport & metabolism
S-adenosyl-methyltransferase MraW	1471	Translation
S-adenosylmethionine-dependent methyltransferase, YraL family	852	Coenzyme transport & metabolism
S-ribosylhomocysteinase LuxS	542	Coenzyme transport & metabolism
septum formation protein Maf	697	Nucleotide transport & metabolism
septum formation protein Maf	697	Cell division
serine--tRNA ligase	6041	Translation
signal recognition particle protein	2401	Intracellular trafficking
signal recognition particle-docking protein FtsY	310	Intracellular trafficking
single-strand binding family protein	155	Replication, recombination, and repair
sortase family protein	1162	Cell wall/ membrane
Sua5/YciO/YrdC/YwlC family protein	620	Translation
SUF system FeS assembly protein, NifU family	232	Energy production & conversion
sugar-binding domain protein	310	Carbohydrate transport & metabolism
sugar-binding domain protein	774	Transcription
tetrahydrofolate dehydrogenase/cyclohydrolase, NAD(P)-binding domain protein	1394	Nucleotide transport & metabolism
thiamine pyrophosphate enzyme, N-terminal TPP binding domain protein	2556	Coenzyme transport & metabolism
thiazole biosynthesis protein ThiG	155	Coenzyme transport & metabolism
Thiol-activated cytolysin	3330	Cell wall/ membrane
thioredoxin	155	Posttranslational modification
threonine synthase	2633	Amino acid transport & metabolism

threonine--tRNA ligase	697	Translation
threonine--tRNA ligase	5034	Translation
thymidylate synthase	1162	Nucleotide transport & metabolism
TIM-barrel protein, nifR3 family	1239	Translation
TRAM domain protein	929	Translation
transaldolase	1084	Coenzyme transport & metabolism
transcription antitermination factor NusB	310	Transcription
transcription termination factor NusA	1162	Transcription
transcription termination factor Rho	310	Transcription
transcription termination factor Rho	1704	Transcription
transcription termination/antitermination factor NusG	310	Transcription
transcription-repair coupling factor	697	Replication, recombination, and repair
transcriptional regulator NrdR	542	Transcription
transcriptional regulator, AsnC family	542	Transcription
transcriptional regulator, DeoR family	774	Transcription
transcriptional regulator, Fur family	232	Inorganic ion transport & metabolism
transcriptional regulator, GntR family	697	Transcription
transcriptional regulator, LacI family	232	Transcription
transcriptional regulator, LuxR family	232	Transcription
transcriptional regulator, LysR family	155	Transcription
transcriptional regulator, MerR family	154	Transcription
transcriptional regulator, Sir2 family	465	Transcription
transcriptional regulatory protein, C-terminal domain protein	232	Signal transduction mechanisms
transcriptional regulatory protein, C-terminal domain protein	232	Transcription
transglycosylase	2866	Cell wall/ membrane
transketolase	18510	Coenzyme transport & metabolism
translation elongation factor G	465	Translation
translation elongation factor G	12159	Translation

translation elongation factor Ts	155	Translation
translation elongation factor Tu	2866	Translation
translation elongation factor Tu	47165	Translation
translation initiation factor IF-2	3098	Translation
translation initiation factor IF-3	155	Translation
transporter, major facilitator family protein	155	Inorganic ion transport & metabolism
transporter, major facilitator family protein	155	Carbohydrate transport & metabolism
transporter, major facilitator family protein	155	Amino acid transport & metabolism
transporter, major intrinsic protein (MIP) family protein	232	Intracellular trafficking
trigger factor	5499	Cell division
triose-phosphate isomerase	387	Carbohydrate transport & metabolism
tRNA (guanine-N(1)-)-methyltransferase	929	Translation
tRNA (guanine-N(7)-)-methyltransferase	231	Translation
tRNA adenylyltransferase	231	Translation
tRNA adenylyltransferase	2711	Translation
tRNA nucleotidyltransferase	155	Translation
tRNA pseudouridine synthase A	155	Translation
tRNA pseudouridine synthase B	232	Translation
tRNA-guanine transglycosylase	1936	Nucleotide transport & metabolism
trypsin	154	Posttranslational modification
tryptophan--tRNA ligase	4260	Translation
tyrosine--tRNA ligase	4260	Translation
UDP-galactopyranose mutase	1394	Cell wall/ membrane
UDP-glucose 4-epimerase	2478	Cell wall/ membrane
UDP-N-acetylglucosamine 1-carboxyvinyltransferase	1162	Cell wall/ membrane
UDP-N-acetylmuramate--L-alanine ligase	465	Cell wall/ membrane
UDP-N-acetylmuramoyl-L-alanine--D-glutamate ligase	774	Cell wall/ membrane
UDP-N-acetylmuramoyl-tripeptide--D-alanyl-D- alanine ligase	1239	Cell wall/ membrane

UMP kinase	1781	Nucleotide transport & metabolism
universal bacterial protein YeaZ	387	Posttranslational modification
universal stress family protein	542	Signal transduction mechanisms
uracil phosphoribosyltransferase	4414	Nucleotide transport & metabolism
uracil-DNA glycosylase	697	Replication, recombination, and repair
UTP--glucose-1-phosphate uridylyltransferase	308	Carbohydrate transport & metabolism
UTP--hexose-1-phosphate uridylyltransferase	852	Energy production & conversion
UvrD/REP helicase	1549	Replication, recombination, and repair
valine--tRNA ligase	7048	Translation
xanthine phosphoribosyltransferase	465	Nucleotide transport & metabolism
YceG family protein	154	Translation

Supplemental Table 3. List of proteins, their abundance, and their associated functional pathway identified in *L. crispatus* bEVs.

Protein Name	Abundance (ppm)	Functional pathway
[citrate (pro-3S)-lyase] ligase	704	Coenzyme transport & metabolism
1-phosphofructokinase	1816	Coenzyme transport & metabolism
16S rRNA methyltransferase GidB	1089	Translation
23S rRNA (uracil-5-)-methyltransferase RumA	1759	Translation
30S ribosomal protein S13	726	Translation
30S ribosomal protein S4	1816	Translation
4-phosphoerythronate dehydrogenase	3268	Coenzyme transport & metabolism
4-phosphoerythronate dehydrogenase	3268	Energy production & conversion
5'-nucleotidase, C-terminal domain protein	1816	Nucleotide transport & metabolism
5'-nucleotidase, C-terminal domain protein	2179	Nucleotide transport & metabolism
50S ribosomal protein L19	726	Translation
50S ribosomal protein L3	1089	Translation
50S ribosomal protein L4	1089	Translation
6-phosphofructokinase	2905	Nucleotide transport & metabolism
6-phosphogluconate dehydrogenase, decarboxylating	726	Coenzyme transport & metabolism
ABC transporter glutamine-binding protein GlnH	1056	Signal transduction mechanisms
ABC transporter glutamine-binding protein GlnH	1056	Amino acid transport & metabolism
ABC transporter, solute-binding protein	1089	Carbohydrate transport & metabolism
ABC transporter, solute-binding protein	4720	Carbohydrate transport & metabolism
ABC transporter, substrate-binding protein	2542	Inorganic ion transport & metabolism
ABC transporter, substrate-binding protein, family 5	726	Amino acid transport & metabolism
ABC transporter, substrate-binding protein, family 5	1089	Amino acid transport & metabolism
ABC transporter, substrate-binding protein, family 5	1089	Amino acid transport & metabolism
acetyl-CoA C-acetyltransferase	2179	Lipid transport & metabolism

acyl carrier protein	726	Secondary metabolites biosynthesis, transport and catabolism
acyl carrier protein	726	Lipid transport & metabolism
Acyl-ACP thioesterase	726	Lipid transport & metabolism
adenine phosphoribosyltransferase	2905	Nucleotide transport & metabolism
adenylate kinase	726	Nucleotide transport & metabolism
adenylosuccinate synthase	3268	Nucleotide transport & metabolism
alanine racemase	2542	Amino acid transport & metabolism
alanine--tRNA ligase	5447	Translation
aldehyde dehydrogenase (NAD) family protein	5810	Energy production & conversion
aldose 1-epimerase	2179	Carbohydrate transport & metabolism
aminopeptidase E	1816	Amino acid transport & metabolism
aminopeptidase N	2542	Amino acid transport & metabolism
aminotransferase, class V	726	Amino acid transport & metabolism
aromatic amino acid aminotransferase	6173	Amino acid transport & metabolism
aspartate racemase	1056	Cell wall/ membrane
aspartate--tRNA ligase	1089	Translation
ATP synthase F1, alpha subunit	726	Energy production & conversion
ATP synthase F1, beta subunit	1089	Energy production & conversion
ATP-dependent Clp endopeptidase, proteolytic subunit ClpP	2905	Posttranslational modification
ATP-dependent protease HslVU, peptidase subunit	2179	Posttranslational modification
ATPase family associated with various cellular activities (AAA)	704	Posttranslational modification
ATPase family associated with various cellular activities (AAA)	2542	Posttranslational modification
BadF/BadG/BcrA/BcrD ATPase family protein	726	Carbohydrate transport & metabolism
cadmium-exporting ATPase	726	Inorganic ion transport & metabolism
cell division protein FtsA	2905	Cell division
cell division protein FtsZ	726	Cell division
cell shape determining protein, MreB/Mrl family	1089	Cell division

chaperone protein DnaK	5810	Posttranslational modification
chaperonin GroL	1089	Posttranslational modification
citrate lyase, alpha subunit	704	Coenzyme transport & metabolism
citrate lyase, beta subunit	1089	Carbohydrate transport & metabolism
Creatinase	5810	Amino acid transport & metabolism
cysteine--tRNA ligase	1816	Translation
cysteine--tRNA ligase	2542	Translation
D-ala D-ala ligase N-terminal domain protein	2179	Nucleotide transport & metabolism
D-alanine--poly(phosphoribitol) ligase, subunit 1	726	Coenzyme transport & metabolism
D-alanine--poly(phosphoribitol) ligase, subunit 2	726	Translation
D-lactate dehydrogenase	8351	Energy production & conversion
D-xylulose 5-phosphate/D-fructose 6-phosphate phosphoketolase	9441	Carbohydrate transport & metabolism
D-xylulose 5-phosphate/D-fructose 6-phosphate phosphoketolase	16703	Carbohydrate transport & metabolism
D-xylulose 5-phosphate/D-fructose 6-phosphate phosphoketolase	17429	Carbohydrate transport & metabolism
deoxyadenosine kinase	726	Nucleotide transport & metabolism
DHHA2 domain protein	3994	Energy production & conversion
dihydrofolate reductase	652	Coenzyme transport & metabolism
dipeptidase PepV	1452	Amino acid transport & metabolism
dipeptidase PepV	1452	Amino acid transport & metabolism
DNA polymerase III, beta subunit	3268	Replication, recombination, and repair
DNA-directed RNA polymerase subunit beta	1089	Transcription
DNA-directed RNA polymerase, alpha subunit	2542	Transcription
DNA-directed RNA polymerase, alpha subunit	3268	Transcription
DNA-directed RNA polymerase, beta subunit	726	Transcription
dTMP kinase	1452	Nucleotide transport & metabolism
elongation factor 4	1452	Cell wall/ membrane
exonuclease, DNA polymerase III, epsilon subunit family	2542	Replication, recombination, and repair
fatty acid/phospholipid synthesis protein PlsX	3994	Lipid transport & metabolism

formate--tetrahydrofolate ligase	1816	Nucleotide transport & metabolism
fructose-bisphosphate aldolase class-II	2905	Carbohydrate transport & metabolism
gametolysin	726	Cell wall/ membrane
glucan 1,6-alpha-glucosidase	1452	Carbohydrate transport & metabolism
glucosamine-6-phosphate deaminase	1452	Carbohydrate transport & metabolism
glutamate--tRNA ligase	1629	Translation
glutamine synthetase, type I	2905	Amino acid transport & metabolism
glutamine-fructose-6-phosphate transaminase (isomerizing)	3994	Cell wall/ membrane
glyceraldehyde-3-phosphate dehydrogenase, type I	5084	Carbohydrate transport & metabolism
glycerol kinase	652	Nucleotide transport & metabolism
glycerol-3-phosphate cytidylyltransferase	726	Cell wall/ membrane
glycerol-3-phosphate cytidylyltransferase	726	Lipid transport & metabolism
glycine hydroxymethyltransferase	652	Amino acid transport & metabolism
glycine hydroxymethyltransferase	978	Amino acid transport & metabolism
glycine--tRNA ligase, alpha subunit	2542	Translation
glycine--tRNA ligase, beta subunit	8715	Translation
glycosyl hydrolase family 25	1816	Cell wall/ membrane
glycosyl hydrolase, family 1	704	Carbohydrate transport & metabolism
Gram-positive signal peptide protein, YSIRK family	1089	Cell division
Gram-positive signal peptide protein, YSIRK family	1452	Cell wall/ membrane
GTP-binding protein TypA	10530	Signal transduction mechanisms
GTP-binding protein YchF	7262	Translation
histidine triad domain protein	2905	Carbohydrate transport & metabolism
histidine triad domain protein	2905	Nucleotide transport & metabolism
histidine--tRNA ligase	11619	Translation
homoserine dehydrogenase	1759	Amino acid transport & metabolism
hydrolase, NUDIX family	1452	Replication, recombination, and repair
hydroxymethylglutaryl-CoA synthase	1452	Lipid transport & metabolism

hydroxymethylglutaryl-CoA synthase	1452	Lipid transport & metabolism
hypothetical protein	726	Carbohydrate transport & metabolism
hypothetical protein	1089	Transcription
hypothetical protein	1089	Carbohydrate transport & metabolism
hypothetical protein	1759	Nucleotide transport & metabolism
hypothetical protein	1955	Replication, recombination, and repair
hypothetical protein	2179	Translation
hypothetical protein	3631	Lipid transport & metabolism
hypothetical protein	7625	Amino acid transport & metabolism
hypothetical protein	7988	Amino acid transport & metabolism
hypoxanthine phosphoribosyltransferase	1452	Nucleotide transport & metabolism
iojap-like protein	726	Translation
isoleucine--tRNA ligase	16340	Translation
L-lactate dehydrogenase	5084	Energy production & conversion
leucine--tRNA ligase	9078	Translation
lipid kinase, YegS/Rv2252/BmrU family	1452	Carbohydrate transport & metabolism
lipid kinase, YegS/Rv2252/BmrU family	1452	Carbohydrate transport & metabolism
low molecular weight phosphotyrosine protein phosphatase	1452	Signal transduction mechanisms
lysine--tRNA ligase	7625	Translation
lysine--tRNA ligase	10893	Translation
M42 glutamyl aminopeptidase	704	Amino acid transport & metabolism
membrane alanyl aminopeptidase	3268	Amino acid transport & metabolism
methionine adenosyltransferase	1452	Coenzyme transport & metabolism
methionine aminopeptidase, type I	3268	Amino acid transport & metabolism
methionine aminopeptidase, type I	3268	Amino acid transport & metabolism
methionine--tRNA ligase	2542	Translation
methionyl-tRNA formyltransferase	978	Translation
methylenetetrahydrofolate dehydrogenase/methenyltetrahydrofolate cyclohydrolase	2542	Nucleotide transport & metabolism

methylenetetrahydrofolate-tRNA-(uracil-5-)- methyltransferase TrmFO	1816	Translation
MTA/SAH nucleosidase	2179	Amino acid transport & metabolism
Mur ligase middle domain protein	726	Cell wall/ membrane
nitroreductase family protein	1452	Energy production & conversion
NlpC/P60 family protein	1452	Cell wall/ membrane
nrdI protein	1452	Nucleotide transport & metabolism
oligoendopeptidase F	1452	Amino acid transport & metabolism
oligoendopeptidase F	2905	Amino acid transport & metabolism
orotidine 5'-phosphate decarboxylase/HUMPS family	2905	Carbohydrate transport & metabolism
oxidoreductase, aldo/keto reductase family protein	9078	Energy production & conversion
penicillin-binding protein, 1A family	652	Cell wall/ membrane
peptidase C1-like family	1452	Amino acid transport & metabolism
peptidase C1-like family	1816	Amino acid transport & metabolism
peptidase family M13	726	Posttranslational modification
peptidase T	1089	Amino acid transport & metabolism
peptide chain release factor 1	6173	Translation
peptide chain release factor 2	726	Translation
peptide chain release factor 3	1089	Translation
peptide deformylase	1089	Translation
phenylalanine--tRNA ligase, beta subunit	2542	Translation
phosphate acetyltransferase	1816	Energy production & conversion
phosphate/phosphite/phosphonate ABC transporter, periplasmic binding protein	1089	Inorganic ion transport & metabolism
phosphoenolpyruvate carboxykinase (GTP)	10167	Coenzyme transport & metabolism
phosphoenolpyruvate carboxykinase (GTP)	11619	Coenzyme transport & metabolism
phosphoenolpyruvate-dependent sugar phosphotransferase system, EIIA 2	704	Carbohydrate transport & metabolism
phosphoenolpyruvate-protein phosphotransferase	7988	Carbohydrate transport & metabolism
phosphoglucomutase/phosphomannomutase, alpha/beta/alpha domain II	2542	Carbohydrate transport & metabolism
phosphoglucosamine mutase	7262	Carbohydrate transport & metabolism

phosphoglycerate kinase	3268	Nucleotide transport & metabolism
phosphoglycerate kinase	3268	Nucleotide transport & metabolism
phosphoglycerate mutase 1 family	6173	Carbohydrate transport & metabolism
phosphopyruvate hydratase	1089	Carbohydrate transport & metabolism
phosphorylase family	2542	Nucleotide transport & metabolism
PPIC-type PPIASE domain protein	3994	Cell wall/ membrane
preprotein translocase, YajC subunit	726	Intracellular trafficking
proline--tRNA ligase	1089	Translation
proline-specific peptidase	5084	Amino acid transport & metabolism
protein-(glutamine-N5) methyltransferase, release factor-specific	3631	Translation
PTS system, mannose/fructose/sorbose family, IIB component	652	Carbohydrate transport & metabolism
PTS system, mannose/fructose/sorbose family, IID component	726	Carbohydrate transport & metabolism
pur operon repressor PurR	1089	Nucleotide transport & metabolism
putative catabolite control protein A	3994	Transcription
putative citrate (pro-3S)-lyase, beta subunit	1452	Carbohydrate transport & metabolism
putative fructose-1,6-bisphosphate aldolase, class II	7262	Carbohydrate transport & metabolism
putative glycerol-3-phosphate dehydrogenase [NAD(P)+]	5447	Lipid transport & metabolism
putative glyoxylate reductase	3268	Coenzyme transport & metabolism
putative glyoxylate reductase	3268	Energy production & conversion
putative GTP-binding protein TypA	9078	Signal transduction mechanisms
putative phosphoglucomutase	2542	Carbohydrate transport & metabolism
putative ribosomal protein S1	5447	Translation
putative RNA methyltransferase, TrmH family, group 2	2179	Translation
putative translation elongation factor G	1089	Translation
pyruvate kinase	36674	Carbohydrate transport & metabolism
pyruvate kinase	36674	Carbohydrate transport & metabolism
pyruvate oxidase	3994	Coenzyme transport & metabolism
pyruvate oxidase	3994	Amino acid transport & metabolism

RecA protein	726	Replication, recombination, and repair
ribonucleoside-diphosphate reductase subunit beta	7262	Nucleotide transport & metabolism
ribonucleoside-diphosphate reductase, alpha subunit	5084	Nucleotide transport & metabolism
ribonucleoside-diphosphate reductase, alpha subunit	5084	Nucleotide transport & metabolism
ribonucleoside-diphosphate reductase, beta subunit	6536	Nucleotide transport & metabolism
ribose-phosphate diphosphokinase	1452	Nucleotide transport & metabolism
ribosomal protein L1	3994	Translation
ribosomal protein L10	2542	Translation
ribosomal protein L11	726	Translation
ribosomal protein L11 methyltransferase	1452	Translation
ribosomal protein L13	1089	Translation
ribosomal protein L14	1089	Translation
ribosomal protein L18	1089	Translation
ribosomal protein L20	726	Translation
ribosomal protein L21	704	Translation
ribosomal protein L22	1816	Translation
ribosomal protein L23	1452	Translation
ribosomal protein L5	2542	Translation
ribosomal protein L7/L12	2905	Translation
ribosomal protein S10	1089	Translation
ribosomal protein S2	3631	Translation
ribosomal protein S3	726	Translation
ribosomal protein S5	1816	Translation
ribosomal protein S7	2179	Translation
ribosomal protein S8	1089	Translation
ribosomal subunit interface protein	726	Translation
RNA polymerase sigma factor RpoD	1089	Transcription
S-ribosylhomocysteinase LuxS	3268	Coenzyme transport & metabolism

serine protease do-like htrA	726	Posttranslational modification
serine--tRNA ligase	3631	Translation
serine-type D-Ala-D-Ala carboxypeptidase	704	Cell wall/ membrane
serine-type D-Ala-D-Ala carboxypeptidase	1056	Cell wall/ membrane
signal recognition particle protein	2905	Intracellular trafficking
single-strand binding family protein	704	Replication, recombination, and repair
Sua5/YciO/YrdC/YwlC family protein	1816	Translation
thiamine biosynthesis/tRNA modification protein ThiI	3268	Coenzyme transport & metabolism
threonine synthase	3994	Amino acid transport & metabolism
threonine--tRNA ligase	8715	Translation
threonine--tRNA ligase	9441	Translation
thymidine kinase	1452	Nucleotide transport & metabolism
thymidylate synthase	2905	Nucleotide transport & metabolism
thymidylate synthase	3268	Nucleotide transport & metabolism
transcription termination factor NusA	7262	Transcription
translation elongation factor G	21786	Translation
translation elongation factor Ts	1816	Translation
triacylglycerol lipase	3631	Lipid transport & metabolism
trigger factor	5810	Cell division
triose-phosphate isomerase	11983	Carbohydrate transport & metabolism
tRNA (5-methylaminomethyl-2-thiouridylate)-methyltransferase	726	Translation
tRNA (guanine-N(1)-)-methyltransferase	726	Translation
tRNA (guanine-N(1)-)-methyltransferase	726	Translation
tRNA (guanine-N(7)-)-methyltransferase	726	Translation
tRNA nucleotidyltransferase/poly(A) polymerase family protein	1089	Translation
tRNA pseudouridine synthase B	652	Translation
tRNA uridine 5-carboxymethylaminomethyl modification enzyme GidA	1089	Cell division
tryptophan--tRNA ligase	704	Translation

tyrosine--tRNA ligase	3268	Translation
UDP-glucose 4-epimerase	3994	Cell wall/ membrane
UDP-N-acetylglucosamine diphosphorylase/glucosamine-1-phosphate N-acetyltransferase	3268	Cell wall/ membrane
UDP-N-acetylmuramate--alanine ligase	1452	Cell wall/ membrane
UDP-N-acetylmuramate--L-alanine ligase	1452	Cell wall/ membrane
UDP-N-acetylmuramate--L-alanine ligase	2542	Cell wall/ membrane
UDP-N-acetylmuramoyl-L-alanine--D-glutamate ligase	1089	Cell wall/ membrane
UDP-N-acetylmuramoyl-L-alanyl-D-glutamate--2, 6-diaminopimelate ligase	726	Cell wall/ membrane
UDP-N-acetylmuramoyl-tripeptide--D-alanyl-D- alanine ligase	2542	Cell wall/ membrane
UDP-N-acetylmuramoyl-tripeptide--D-alanyl-D- alanine ligase	3268	Cell wall/ membrane
UMP kinase	1089	Nucleotide transport & metabolism
undecaprenyldiphospho-muramoylpentapeptide beta-N-acetylglucosaminyltransferase	726	Cell wall/ membrane
universal stress family protein	1816	Signal transduction mechanisms
uracil phosphoribosyltransferase	2542	Nucleotide transport & metabolism
UTP--glucose-1-phosphate uridylyltransferase	1452	Cell wall/ membrane
valine--tRNA ligase	6899	Translation
Xaa-Pro dipeptidase	5447	Amino acid transport & metabolism
Xaa-Pro dipeptidyl-peptidase	652	Amino acid transport & metabolism
YSIRK family Gram-positive signal peptide	1452	Cell wall/ membrane

Supplemental Table 4. List of proteins, their abundance, and their associated functional pathway identified in *M. mulieris* bEVs.

Protein Name	Abundance (ppm)	Functional pathway
1-deoxy-D-xylulose 5-phosphate reductoisomerase	493	Lipid transport & metabolism
1,4-alpha-glucan branching enzyme	1150	Carbohydrate transport & metabolism
3-dehydroquinate synthase	1807	Energy production & conversion
30S ribosomal protein S1	4763	Translation
30S ribosomal protein S13	425	Translation
30S ribosomal protein S17	329	Translation
4-alpha-glucanotransferase	2792	Carbohydrate transport & metabolism
5-methyltetrahydropteroyltriglutamate-- homocysteine S-methyltransferase	6242	Coenzyme transport & metabolism
50S ribosomal protein L3	599	Translation
50S ribosomal protein L4	493	Translation
6-phosphofructokinase	1643	Carbohydrate transport & metabolism
ABC transporter, ATP-binding protein	329	Defense mechanisms
ABC transporter, ATP-binding protein	599	Amino acid transport & metabolism
ABC transporter, ATP-binding protein	1150	Carbohydrate transport & metabolism
ABC transporter, ATP-binding protein	6406	Inorganic ion transport & metabolism
ABC transporter, permease protein	329	Inorganic ion transport & metabolism
ABC transporter, solute-binding protein	16590	Carbohydrate transport & metabolism
ABC transporter, substrate-binding protein	1971	Inorganic ion transport & metabolism
ABC transporter, substrate-binding protein, family 3	4106	Signal transduction mechanisms
ABC transporter, substrate-binding protein, family 3	4106	Amino acid transport & metabolism
ABC transporter, substrate-binding protein, family 5	821	Amino acid transport & metabolism
ABC transporter, substrate-binding protein, thiB family	1478	Coenzyme transport & metabolism
acetate kinase	3778	Coenzyme transport & metabolism
acetyl xylan esterase (AXE1)	599	Secondary metabolites biosynthesis, transport and catabolism

adenylate kinase	1807	Nucleotide transport & metabolism
adenylosuccinate synthase	2300	Nucleotide transport & metabolism
alanine racemase	1643	Cell wall/ membrane
alanine--tRNA ligase	2135	Translation
alpha amylase, catalytic domain protein	1971	Carbohydrate transport & metabolism
alpha-glucan phosphorylase	23982	Carbohydrate transport & metabolism
aminotransferase, class I/II	2792	Amino acid transport & metabolism
aminotransferase, class III	799	Amino acid transport & metabolism
AMP-binding enzyme	1198	Lipid transport & metabolism
Anticodon-binding domain protein	2300	Translation
AP endonuclease, family 2	986	Carbohydrate transport & metabolism
apolipoprotein N-acyltransferase	399	Cell wall/ membrane
arabinose isomerase	1314	Carbohydrate transport & metabolism
arginine--tRNA ligase	657	Translation
argininosuccinate lyase	329	Amino acid transport & metabolism
argininosuccinate synthase	2135	Amino acid transport & metabolism
aspartate--tRNA ligase	986	Translation
aspartyl/glutamyl-tRNA(Asn/Gln) amidotransferase, A subunit	1478	Translation
aspartyl/glutamyl-tRNA(Asn/Gln) amidotransferase, B subunit	4763	Translation
ATP synthase F1, delta subunit	657	Energy production & conversion
ATP synthase F1, gamma subunit	1643	Energy production & conversion
ATP-dependent chaperone protein ClpB	3121	Posttranslational modification
ATP-dependent metallopeptidase HflB	329	Posttranslational modification
bacterial cytochrome ubiquinol oxidase	638	Energy production & conversion
bacterial flagellin domain protein	1643	Cell motility
branched-chain amino acid ABC transporter, permease protein	329	Carbohydrate transport & metabolism
branched-chain-amino-acid transaminase	2628	Coenzyme transport & metabolism
branched-chain-amino-acid transaminase	2628	Amino acid transport & metabolism

BRO family, N-terminal domain protein	329	Transcription
carbohydrate kinase, FGGY family protein	821	Carbohydrate transport & metabolism
cell envelope-like function transcriptional attenuator common domain protein	493	Transcription
chaperone protein DnaK	493	Posttranslational modification
chaperonin GroL	2957	Posttranslational modification
chemotaxis response regulator protein-glutamate methylesterase of group 1 operon	1478	Signal transduction mechanisms
chemotaxis response regulator protein-glutamate methylesterase of group 1 operon	1478	Cell motility
CheR methyltransferase, SAM binding domain protein	657	Signal transduction mechanisms
CheR methyltransferase, SAM binding domain protein	657	Cell motility
CheW-like protein	3121	Signal transduction mechanisms
CRISPR system CASCADE complex protein CasC	329	Replication, recombination, and repair
cysteine desulfurase, SufS subfamily	1150	Amino acid transport & metabolism
cysteine synthase A	4106	Amino acid transport & metabolism
cysteine--tRNA ligase	2628	Translation
cytosol aminopeptidase family, catalytic domain protein	2300	Amino acid transport & metabolism
D-ala D-ala ligase N-terminal domain protein	657	Nucleotide transport & metabolism
D-alanyl-D-alanine carboxypeptidase/D-alanyl-D-alanine-endopeptidase	986	Cell wall/ membrane
DegT/DnrJ/EryC1/StrS aminotransferase family protein	2135	Amino acid transport & metabolism
diaminopimelate decarboxylase	799	Amino acid transport & metabolism
dihydrodipicolinate synthase	329	Amino acid transport & metabolism
dihydrolipoyl dehydrogenase	399	Energy production & conversion
dihydroxyacetone kinase, DhaK subunit	1971	Carbohydrate transport & metabolism
dihydroxyacetone kinase, L subunit	329	Carbohydrate transport & metabolism
DivIVA domain repeat protein	1150	Cell division
DNA (cytosine-5-)-methyltransferase	1150	Replication, recombination, and repair
DNA gyrase, A subunit	493	Replication, recombination, and repair
DNA polymerase III, beta subunit	657	Replication, recombination, and repair
DNA-binding regulatory protein, YebC/PmpR family	599	Transcription

DsbA-like protein	657	Posttranslational modification
dTDP-4-dehydrorhamnose reductase	1150	Cell wall/ membrane
dTDP-glucose 4,6-dehydratase	3285	Cell wall/ membrane
dTMP kinase	399	Nucleotide transport & metabolism
efflux ABC transporter, permease protein	399	Defense mechanisms
endopeptidase Clp	425	Intracellular trafficking
endopeptidase Clp	425	Posttranslational modification
exonuclease	821	Replication, recombination, and repair
export membrane protein SecD	329	Intracellular trafficking
export membrane protein SecF	399	Intracellular trafficking
FAH family protein	329	Secondary metabolites biosynthesis, transport and catabolism
FemAB family protein	799	Defense mechanisms
ferritin-like protein	2300	Inorganic ion transport & metabolism
ferrochelatase	821	Coenzyme transport & metabolism
FeS assembly ATPase SufC	1643	Posttranslational modification
FeS assembly protein SufB	2135	Posttranslational modification
FeS assembly protein SufD	329	Posttranslational modification
FGGY-family pentulose kinase	329	Carbohydrate transport & metabolism
FHA domain protein	399	Signal transduction mechanisms
fimbrial subunit type 1	821	Cell wall/ membrane
flagellar basal-body rod protein FlgC	399	Cell motility
flagellar hook-basal body protein	2300	Cell motility
flagellar motor switch protein FliN	329	Cell motility
formyltetrahydrofolate deformylase	799	Nucleotide transport & metabolism
fructose-bisphosphate aldolase, class II	5420	Carbohydrate transport & metabolism
FTR1 family protein	329	Inorganic ion transport & metabolism
fumarate hydratase, class II	3614	Energy production & conversion

GDSL-like protein	657	Amino acid transport & metabolism
Glu/Leu/Phe/Val dehydrogenase, dimerization domain protein	7227	Amino acid transport & metabolism
glucose-1-phosphate adenylyltransferase	329	Coenzyme transport & metabolism
glucose-1-phosphate thymidylyltransferase	2628	Coenzyme transport & metabolism
glutamate--tRNA ligase	1314	Translation
glutamate-1-semialdehyde-2,1-aminomutase	657	Coenzyme transport & metabolism
glutamine synthetase, type I	2397	Amino acid transport & metabolism
glutamine-dependent NAD(+) synthetase domain protein	986	Coenzyme transport & metabolism
glutamine-fructose-6-phosphate transaminase (isomerizing)	799	Cell wall/ membrane
glyceraldehyde-3-phosphate dehydrogenase, type I	12155	Carbohydrate transport & metabolism
glycerate kinase	986	Carbohydrate transport & metabolism
glycerol kinase	493	Nucleotide transport & metabolism
glycerol-3-phosphate dehydrogenase, anaerobic, A subunit	329	Energy production & conversion
glycerol-3-phosphate dehydrogenase, anaerobic, B subunit	2135	Amino acid transport & metabolism
glycine hydroxymethyltransferase	3121	Amino acid transport & metabolism
glycine--tRNA ligase	1807	Translation
glycogen debranching enzyme GlgX	493	Carbohydrate transport & metabolism
glycogen synthase, Corynebacterium family	599	Carbohydrate transport & metabolism
glycosyl hydrolase, family 31	329	Carbohydrate transport & metabolism
glycosyltransferase, group 1 family protein	657	Cell wall/ membrane
GMP synthase (glutamine-hydrolyzing) domain protein	399	Nucleotide transport & metabolism
GroES-like protein	493	Energy production & conversion
GTP cyclohydrolase I	399	Nucleotide transport & metabolism
GTP-binding protein LepA	599	Cell wall/ membrane
HAD hydrolase, family IIA	1478	Carbohydrate transport & metabolism
helicase C-terminal domain protein	329	Replication, recombination, and repair
helicase C-terminal domain protein	329	Transcription
histidine triad domain protein	657	Carbohydrate transport & metabolism

histidine triad domain protein	657	Nucleotide transport & metabolism
histidine--tRNA ligase	2135	Translation
homoserine dehydrogenase	329	Amino acid transport & metabolism
hypothetical protein	799	Replication, recombination, and repair
hypothetical protein	821	Cell wall/ membrane
hypothetical protein	986	Translation
hypothetical protein	986	Coenzyme transport & metabolism
hypothetical protein	1150	Posttranslational modification
hypothetical protein	2300	Cell motility
hypothetical protein	2464	Carbohydrate transport & metabolism
hypothetical protein	2957	Inorganic ion transport & metabolism
hypothetical protein	5420	Cytoskeleton
hypothetical protein	5420	Cell division
hypothetical protein	5992	Defense mechanisms
hypoxanthine phosphoribosyltransferase	329	Nucleotide transport & metabolism
inorganic diphosphatase	329	Energy production & conversion
iron dependent repressor DNA binding domain protein	1478	Transcription
isocitrate dehydrogenase, NADP-dependent	986	Energy production & conversion
isoleucine--tRNA ligase	2792	Translation
kinase domain protein	986	Signal transduction mechanisms
kinase domain protein	986	Replication, recombination, and repair
kinase domain protein	986	Transcription
L-lactate dehydrogenase	2300	Energy production & conversion
lactaldehyde reductase	5913	Energy production & conversion
leucine--tRNA ligase	4435	Translation
Lon protease (S16) C-terminal proteolytic domain protein	493	Signal transduction mechanisms
LPXTG-motif cell wall anchor domain protein	7720	Intracellular trafficking
LPXTG-motif cell wall anchor domain protein	7720	Cell motility

malate dehydrogenase	3285	Energy production & conversion
mannose-6-phosphate isomerase, class I	493	Carbohydrate transport & metabolism
methionine adenosyltransferase	2300	Coenzyme transport & metabolism
methionine aminopeptidase, type I	657	Amino acid transport & metabolism
methionine--tRNA ligase	1643	Translation
methionyl-tRNA formyltransferase	329	Translation
methyl-accepting chemotaxis protein signaling domain protein	329	Signal transduction mechanisms
methyl-accepting chemotaxis protein signaling domain protein	329	Cell motility
MgtE intracellular domain protein	329	Inorganic ion transport & metabolism
MIP family channel protein	599	Intracellular trafficking
N-acetylmuramoyl-L-alanine amidase	8049	Cell wall/ membrane
N-acylglucosamine 2-epimerase	493	Carbohydrate transport & metabolism
Na+/H+ antiporter NhaA	329	Inorganic ion transport & metabolism
NAD dependent epimerase/dehydratase family protein	399	Cell wall/ membrane
NAD-dependent glycerol-3-phosphate dehydrogenase C-terminal domain protein	399	Lipid transport & metabolism
NAD(P)(+) transhydrogenase (AB-specific), alpha subunit	399	Energy production & conversion
NADH dehydrogenase (quinone), G subunit	657	Energy production & conversion
NADH dehydrogenase subunit D	399	Energy production & conversion
nicotinate phosphoribosyltransferase	1971	Coenzyme transport & metabolism
nitroreductase family protein	1643	Energy production & conversion
NLPA lipoprotein	4271	Cell wall/ membrane
NlpC/P60 family protein	1478	Cell wall/ membrane
NMT1/THI5-like protein	657	Inorganic ion transport & metabolism
non-canonical purine NTP pyrophosphatase, RdgB/HAM1 family	329	Nucleotide transport & metabolism
nucleotide sugar dehydrogenase	821	Cell wall/ membrane
nucleotidyl transferase	1478	Cell wall/ membrane
O-acetylhomoserine aminocarboxypropyltransferase/cysteine synthase	1314	Amino acid transport & metabolism

O-succinylbenzoate-CoA ligase family protein	329	Secondary metabolites biosynthesis, transport and catabolism
O-succinylbenzoate-CoA ligase family protein	329	Lipid transport & metabolism
orotate phosphoribosyltransferase	493	Nucleotide transport & metabolism
orotidine 5'-phosphate decarboxylase	986	Nucleotide transport & metabolism
oxidoreductase, aldo/keto reductase family protein	329	Energy production & conversion
oxidoreductase, FAD/FMN-binding protein	1598	Energy production & conversion
oxoglutarate dehydrogenase (succinyl-transferring), E1 component	1314	Energy production & conversion
penicillin-binding protein, transpeptidase domain protein	986	Cell wall/ membrane
peptidase, M23 family	6078	Cell wall/ membrane
peptide deformylase	329	Translation
peptidyl-prolyl cis-trans isomerase B	986	Carbohydrate transport & metabolism
peptidyl-prolyl cis-trans isomerase, FKBP-type	4106	Cell wall/ membrane
peptidyl-prolyl cis-trans isomerase, FKBP-type	5420	Posttranslational modification
periplasmic binding protein	821	Inorganic ion transport & metabolism
permease, cytosine/purine, uracil, thiamine, allantoin family	425	Intracellular trafficking
phenylalanine--tRNA ligase, alpha subunit	1314	Translation
phenylalanine--tRNA ligase, beta subunit	3121	Translation
phosphate acetyltransferase	1807	Energy production & conversion
phosphoenolpyruvate carboxykinase (GTP)	30223	Coenzyme transport & metabolism
phosphoenolpyruvate-protein phosphotransferase	329	Carbohydrate transport & metabolism
Phosphofructokinase	1314	Coenzyme transport & metabolism
phosphoglucomutase, alpha-D-glucose phosphate-specific	5913	Carbohydrate transport & metabolism
phosphoglucomutase/phosphomannomutase, alpha/beta/alpha domain II	3121	Carbohydrate transport & metabolism
phosphoglucosamine mutase	1643	Carbohydrate transport & metabolism
phosphoglycerate kinase	3121	Nucleotide transport & metabolism
phosphoglycerate mutase 1 family	7392	Carbohydrate transport & metabolism
phosphoglycerate mutase family protein	399	Carbohydrate transport & metabolism

phosphopyruvate hydratase	986	Coenzyme transport & metabolism
phosphoribosylaminoimidazole carboxylase, ATPase subunit	329	Nucleotide transport & metabolism
phosphoribosylaminoimidazolesuccinocarboxamide synthase	1807	Nucleotide transport & metabolism
phosphoribosylformylglycinamidine synthase I	599	Nucleotide transport & metabolism
phosphoribosylformylglycinamidine synthase II	1807	Nucleotide transport & metabolism
polyphosphate kinase 1	599	Inorganic ion transport & metabolism
polyprenyl synthetase	1398	Coenzyme transport & metabolism
preprotein translocase, SecA subunit	6406	Intracellular trafficking
proline--tRNA ligase	3285	Translation
putative adenine phosphoribosyltransferase	399	Nucleotide transport & metabolism
putative cell wall binding repeat 2	329	Secondary metabolites biosynthesis, transport and catabolism
putative cell wall binding repeat 2	4928	Cytoskeleton
putative cell wall binding repeat 2	4928	Cell division
putative cell wall binding repeat 2	5585	Cell wall/ membrane
putative dTDP-4-dehydrorhamnose 3,5-epimerase	657	Cell wall/ membrane
putative flagellar basal-body rod protein FlgB	329	Cell motility
putative glucosamine-6-phosphate deaminase	986	Carbohydrate transport & metabolism
putative glycoprotease GCP	821	Posttranslational modification
putative HAD hydrolase, TIGR01457 family	1314	Carbohydrate transport & metabolism
putative L-ribulose-5-phosphate 4-epimerase	657	Carbohydrate transport & metabolism
putative N-acetylglucosamine-6-phosphate deacetylase	1643	Carbohydrate transport & metabolism
putative NADH oxidoreductase (quinone), F subunit	493	Energy production & conversion
putative phosphoglycerate dehydrogenase	1478	Coenzyme transport & metabolism
putative phosphoglycerate dehydrogenase	1478	Amino acid transport & metabolism
putative phosphoribosylaminoimidazolecarboxamide formyltransferase/IMP cyclohydrolase	599	Nucleotide transport & metabolism
putative phosphoserine transaminase	329	Amino acid transport & metabolism
pyridine nucleotide-disulfide oxidoreductase	329	Inorganic ion transport & metabolism

pyridoxal 5'-phosphate synthase, synthase subunit Pdx1	425	Coenzyme transport & metabolism
pyrimidine-nucleoside phosphorylase	657	Nucleotide transport & metabolism
pyruvate kinase	5585	Carbohydrate transport & metabolism
pyruvate, phosphate dikinase	12319	Carbohydrate transport & metabolism
response regulator receiver domain protein	399	Signal transduction mechanisms
rhamnan synthesis protein F	399	Cell wall/ membrane
ribonucleoside-diphosphate reductase, beta subunit	599	Nucleotide transport & metabolism
ribose 5-phosphate isomerase	493	Carbohydrate transport & metabolism
ribose-phosphate diphosphokinase	657	Nucleotide transport & metabolism
ribosomal protein L1	986	Translation
ribosomal protein L10	329	Translation
ribosomal protein L13	657	Translation
ribosomal protein L14	329	Translation
ribosomal protein L15	399	Translation
ribosomal protein L16	399	Translation
ribosomal protein L17	1314	Translation
ribosomal protein L19	599	Translation
ribosomal protein L20	399	Translation
ribosomal protein L25, Ctc-form	399	Translation
ribosomal protein L5	657	Translation
ribosomal protein L7/L12	493	Translation
ribosomal protein S10	329	Translation
ribosomal protein S12	399	Translation
ribosomal protein S2	3942	Translation
ribosomal protein S3	1314	Translation
ribosomal protein S4	1971	Translation
ribosomal protein S5	821	Translation
ribosomal subunit interface protein	1971	Translation

ribulokinase	821	Carbohydrate transport & metabolism
ROK family protein	1314	Transcription
ROK family protein	1314	Carbohydrate transport & metabolism
S-adenosyl-methyltransferase MraW	329	Translation
S1 RNA binding domain protein	399	Translation
serine--tRNA ligase	1314	Translation
signal peptidase I	657	Intracellular trafficking
signal peptidase II	599	Intracellular trafficking
signal peptidase II	599	Cell wall/ membrane
signal recognition particle protein	821	Intracellular trafficking
signal recognition particle-docking protein FtsY	657	Intracellular trafficking
SIS domain protein	399	Cell wall/ membrane
Sua5/YciO/YrdC/YwlC family protein	329	Translation
succinate dehydrogenase or fumarate reductase, flavoprotein subunit	2792	Energy production & conversion
succinate-CoA ligase, beta subunit	399	Nucleotide transport & metabolism
sugar-binding domain protein	20039	Carbohydrate transport & metabolism
sugar-phosphate isomerase, RpiB/LacA/LacB family	1150	Carbohydrate transport & metabolism
TAP-like protein	5913	Cell wall/ membrane
Tat pathway signal sequence domain protein	657	Amino acid transport & metabolism
thiamine diphosphokinase	399	Coenzyme transport & metabolism
thiamine pyrophosphate enzyme, central domain protein	599	Amino acid transport & metabolism
thiol reductant ABC exporter, CydC subunit	399	Posttranslational modification
thiol reductant ABC exporter, CydC subunit	399	Energy production & conversion
threonine synthase	986	Amino acid transport & metabolism
transcription termination factor NusA	1150	Transcription
transcriptional regulator, GntR family	799	Transcription
transcriptional regulator, IclR family, C-terminal domain protein	599	Transcription
transglycosylase	5749	Cell wall/ membrane

transketolase	2135	Carbohydrate transport & metabolism
translation elongation factor Tu	13633	Translation
translation initiation factor IF-2	1971	Translation
transporter, lactate permease (LctP) family	599	Energy production & conversion
trigger factor	5256	Cell division
tRNA nucleotidyltransferase	493	Translation
tRNA-guanine transglycosylase	493	Nucleotide transport & metabolism
trypsin	799	Cell wall/ membrane
trypsin	986	Posttranslational modification
tryptophan synthase, beta subunit	2300	Amino acid transport & metabolism
tryptophan--tRNA ligase	821	Translation
type III restriction enzyme, res subunit	399	Replication, recombination, and repair
tyrosine--tRNA ligase	2792	Translation
UbiC transcription regulator-associated domain protein	329	Transcription
UDP-galactopyranose mutase	2628	Cell wall/ membrane
UDP-glucose 4-epimerase	799	Cell wall/ membrane
UDP-N-acetylglucosamine 1-carboxyvinyltransferase	599	Cell wall/ membrane
UDP-N-acetylglucosamine 2-epimerase	1807	Carbohydrate transport & metabolism
UDP-N-acetylglucosamine diphosphorylase/glucosamine-1-phosphate N-acetyltransferase	986	Cell wall/ membrane
UDP-N-acetylmuramoyl-L-alanine--D-glutamate ligase	329	Cell wall/ membrane
UDP-N-acetylmuramoyl-L-alanyl-D-glutamate--2, 6-diaminopimelate ligase	986	Cell wall/ membrane
UDP-N-acetylmuramoyl-tripeptide--D-alanyl-D- alanine ligase	657	Cell wall/ membrane
UMP kinase	657	Nucleotide transport & metabolism
undecaprenyl-diphosphatase UppP	399	Defense mechanisms
undecaprenyldiphospho-muramoylpentapeptide beta-N-acetylglucosaminyltransferase	329	Cell wall/ membrane
uracil phosphoribosyltransferase	1150	Nucleotide transport & metabolism
uroporphyrinogen-III synthase	599	Coenzyme transport & metabolism
UTP--hexose-1-phosphate uridylyltransferase	999	Energy production & conversion

VanW-like protein	2792	Defense mechanisms

Supplemental Table 5. Cytokine expression by cervical and vaginal epithelial cell types after exposure to bEVs derived from *L. crispatus*, *G. vaginalis*, and *M. mulieris* grown in NYC culture medium, expressed as fold-changes relative to NYC culture medium controls

	**Ectocervical**						**Endocervical**						**Vaginal**					
	L. crispatus		*G. vaginalis*		*M. mulieris*		*L. crispatus*		*G. vaginalis*		*M. mulieris*		*L. crispatus*		*G. vaginalis*		*M. mulieris*	
	Fold-change ± SEM	Adjusted p-value	Fold-change ± SEM	Adjusted p-value	Fold-change ± SEM	Adjusted p-value	Fold-change ± SEM	Adjusted p-value	Fold-change ± SEM	Adjusted p-value	Fold-change ± SEM	Adjusted p-value	Fold-change ± SEM	Adjusted p-value	Fold-change ± SEM	Adjusted p-value	Fold-change ± SEM	Adjusted p-value
**G-CSF**	1.05 ± 0.10	ns	5.58 ± 0.51	<0.0001	7.65 ± 0.20	<0.0001	82.67 ± 81.67	ns	78130 ± 14070	<0.0001	73210 ± 3643	<0.0001	1.02 ± 0.06	ns	21.13 ± 2.49	<0.0001	21.47 ± 1.10	<0.0001
**GM-CSF**	0.97 ± 0.06	ns	1.85 ± 0.07	<0.0001	2.47 ± 0.01	<0.0001	1.80 ± 0.07	ns	20.91 ± 2.47	<0.0001	22.09 ± 0.73	<0.0001	0.92 ± 0.03	ns	7.49 ± 0.67	<0.0001	7.88 ± 0.29	<0.0001
**IFNα2**	0.60 ± 0.59	ns	1.50 ± 0.39	ns	2.61 ± 0.96	ns	20.33 ± 19.33	ns	11.00 ± 10.00	ns	93.00 ± 22.30	0.003	1.84 ± 1.79	ns	4.89 ± 2.93	ns	4.44 ± 2.31	ns
**IFNγ**	0.45 ± 0.22	ns	0.80 ± 0.24	ns	0.71 ± 0.22	ns	25.00 ± 14.15	ns	25.33 ± 9.26	ns	55.67 ± 5.67	0.0036	1.38 ± 0.66	ns	1.36 ± 1.00	ns	2.42 ± 1.37	ns
**IL-10**	0.88 ± 0.12	ns	0.97 ± 0.02	ns	0.98 ± 0.13	ns	0.89 ± 0.02	ns	1.13 ± 0.07	ns	1.22 ± 0.14	ns	1.23 ± 0.13	ns	1.07 ± 0.02	ns	1.08 ± 0.04	ns
**IL-12p40**	0.97 ± 0.09	ns	0.66 ± 0.40	ns	1.32 ± 0.34	ns	28.00 ± 8.54	ns	64.67 ± 14.10	0.0298	80.33 ± 18.66	0.0084	1.00 ± 0.0	ns	16.93 ± 15.93	ns	32.87 ± 16.56	ns
**IL-12p70**	0.75 ± 0.16	ns	1.03 ± 0.09	ns	1.08 ± 0.23	ns	1.26 ± 0.61	ns	1.87 ± 0.78	ns	2.11 ± 0.37	ns	3.03 ± 1.48	ns	2.48 ± 0.46	ns	2.62 ± 0.80	ns
**IL-13**	1.87 ± 0.44	ns	4.66 ± 0.21	<0.0001	8.25 ± 0.84	0.0018	1.00 ± 0.0	ns	29.33 ± 2.67	0.0025	16.33 ± 8.95	ns	0.88 ± 0.41	ns	6.81 ± 0.75	ns	7.32 ± 3.69	ns
**IL-15**	0.97 ± 0.11	ns	0.97 ± 0.13	ns	1.14 ± 0.04	ns	1.11 ± 0.02	ns	1.69 ± 0.18	0.0083	1.58 ± 0.15	0.023	1.05 ± 0.16	ns	1.43 ± 0.11	ns	1.46 ± 0.11	ns
**IL-17A**	0.07 ± 0.0	ns	0.93 ± 0.13	ns	1.14 ± 0.90	ns	1.00 ± 0.0	ns	26.67 ± 9.26	0.0289	18.00 ± 7.55	ns	15.40 ± 14.40	ns	15.70 ± 5.99	ns	31.70 ± 4.61	0.0429
**IL-1RA**	0.93 ± 0.14	ns	0.92 ± 0.15	ns	1.16 ± 0.03	ns	0.91 ± 0.03	ns	1.71 ± 0.23	ns	1.58 ± 0.18	ns	0.75 ± 0.11	ns	1.06 ± 0.19	ns	1.32 ± 0.07	ns
**IL-1α**	0.98 ± 0.14	ns	0.98 ± 0.10	ns	1.25 ± 0.04	ns	1.03 ± 0.03	ns	2.19 ± 0.25	0.0061	2.07 ± 0.18	0.0121	0.89 ± 0.14	ns	1.28 ± 0.14	ns	1.61 ± 0.10	ns

**IL-1β**	0.93 ± 0.06	ns	1.08 ± 0.09	ns	1.18 ± 0.03	ns	1.03 ± 0.02	ns	2.78 ± 0.22	<0.0001	2.78 ± 0.22	<0.0001	1.05 ± 0.13	ns	1.51 ± 0.04	0.0049	1.65 ± 0.05	0.0009
**IL-2**	1.01 ± 0.09	ns	0.74 ± 0.09	ns	1.17 ± 0.11	ns	1.10 ± 0.12	ns	1.28 ± 0.19	ns	1.12 ± 0.15	ns	1.68 ± 0.44	ns	1.15 ± 0.14	ns	0.97 ± 0.15	ns
**IL-3**	1.07 ± 0.02	ns	1.18 ± 0.10	ns	1.08 ± 0.15	ns	0.86 ± 0.19	ns	0.79 ± 0.09	ns	1.02 ± 0.15	ns	0.74 ± 0.14	ns	0.40 ± 0.02	0.0023	0.55 ± 0.09	0.0144
**IL-4**	27.33 ± 26.33	ns	1.00 ± 0.0	ns	1.00 ± 0.0	ns	1.00 ± 0.0	ns	2.00 ± 1.00	ns	10.00 ± 9.00	ns	2.59 ± 1.59	ns	1.00 ± 0.0	ns	1.00 ± 0.0	ns
**IL-5**	0.92 ± 0.0	ns	0.98 ± 0.03	ns	0.91 ± 0.07	ns	1.04 ± 0.03	ns	1.02 ± 0.05	ns	1.05 ± 0.09	ns	1.30 ± 0.24	ns	1.18 ± 0.03	ns	1.03 ± 0.01	ns
**IL-6**	1.07 ± 0.10	ns	3.03 ± 0.30	<0.0001	3.90 ± 0.13	<0.0001	1.88 ± 0.03	ns	8.19 ± 1.14	<0.0001	6.45 ± 0.23	<0.0001	0.98 ± 0.11	ns	17.22 ± 1.50	<0.0001	19.35 ± 1.27	<0.0001
**IL-7**	0.93 ± 0.01	ns	1.29 ± 0.05	0.0011	1.43 ± 0.10	0.0137	1.00 ± 0.11	ns	2.74 ± 0.42	0.0024	3.08 ± 0.22	0.0006	0.70 ± 0.20	ns	2.18 ± 0.37	0.0269	2.60 ± 0.13	0.0043
**IL-8**	1.05 ± 0.07	ns	3.93 ± 0.20	<0.0001	5.61 ± 0.24	<0.0001	1.63 ± 0.04	ns	20.17 ± 2.30	<0.0001	20.33 ± 0.72	<0.0001	0.93 ± 0.05	ns	15.13 ± 1.42	<0.0001	16.04 ± 0.78	<0.0001
**IP-10**	1.04 ± 0.11	ns	1.73 ± 0.15	ns	2.36 ± 0.14	ns	1.94 ± 0.20	ns	34.92 ± 5.22	<0.0001	29.34 ± 2.91	<0.0001	0.96 ± 0.06	ns	12.59 ± 1.66	<0.0001	14.71 ± 1.94	0.0001
**MCP-1**	0.96 ± 0.03	ns	1.07 ± 0.05	ns	1.09 ± 0.02	ns	1.03 ± 0.03	ns	1.95 ± 0.13	0.0004	1.85 ± 0.16	0.0002	1.07 ± 0.06	ns	1.14 ± 0.05	ns	1.22 ± 0.04	0.039
**MIP-1α**	0.03 ± 0.02	ns	4.38 ± 0.97	0.0005	7.20 ± 1.14	0.0276	1.00 ± 0.0	ns	23.33 ± 22.33	ns	5.33 ± 4.33	ns	1.00 ± 0.0	ns	1176 ± 159.4	<0.0001	1315.2 ± 84.85	<0.0001
**MIP-1β**	1.00 ± 0.0	ns	48.00 ± 25.36	ns	156.0 ± 46.13	0.003	1.00 ± 0.0	ns	160.7 ± 56.05	0.0046	148.7 ± 13.28	0.0077	1.00 ± 0.0	ns	598.6 ± 85.23	<0.0001	686.9 ± 47.21	<0.0001
**TNFα**	1.17 ± 0.16	ns	5.30 ± 0.50	<0.0001	7.03 ± 0.14	<0.0001	1.23 ± 0.22	ns	9.06 ± 1.10	<0.0001	8.73 ± 0.72	<0.0001	1.35 ± 0.17	ns	15.60 ± 1.68	<0.0001	18.36 ± 1.03	<0.0001
**TNFβ**	0.38 ± 0.17	ns	0.67 ± 0.52	ns	1.05 ± 0.59	ns	1.00 ± 0.0	ns	2.67 ± 1.67	ns	3.00 ± 1.15	ns	8.53 ± 7.53	ns	1.00 ± 0.0	ns	3.40 ± 2.40	ns
**VEGF**	0.87 ± 0.09	ns	0.98 ± 0.14	ns	1.29 ± 0.04	ns	1.11 ± 0.07	ns	2.10 ± 0.32	0.0027	2.02 ± 0.13	0.0047	0.83 ± 0.04	ns	1.29 ± 0.18	ns	1.46 ± 0.02	ns

Statistical analysis was conducted by one-way ANOVA with Tukey's correction for multiple comparisons.

Supplemental Table 6. Cytokine expression by THP-1 monocytes after exposure to bEVs derived from *L. crispatus*, *G. vaginalis*, and *M. mulieris* grown in NYC culture medium, expressed as fold-changes relative to NYC culture medium controls

	L. crispatus		*G. vaginalis*		*M. mulieris*	
	Fold-change ± SEM	Adjusted p-value	Fold-change ± SEM	Adjusted p-value	Fold-change ± SEM	Adjusted p-value
**EGF**	1.10 ± 1.09	ns	11.52 ± 0.35	<0.0001	19.59 ± 0.26	<0.0001
**Eotaxin**	1.00 ± 0.0	ns	334.7 ± 173	ns	761.3 ± 106.0	0.0005
**G-CSF**	0.03 ± 0.0	ns	61.03 ± 4.00	<0.0001	157.0 ± 7.24	<0.0001
**GM-CSF**	1.24 ± 0.12	ns	19.16 ± 2.03	0.0002	41.11 ± 3.58	<0.0001
**IFNα2**	5.67 ± 4.67	ns	123.0 ± 72	ns	192.3 ± 14.67	0.0072
**IFNγ**	6.31 ± 1.24	ns	13.88 ± 0.19	<0.0001	15.58 ± 1.40	<0.0001
**IL-10**	0.38 ± 0.0	ns	67.13 ± 3.95	0.0363	125.8 ± 33.63	0.0006
**IL-12p40**	0.25 ± 0.0	ns	634.9 ± 53.81	0.0007	2733 ± 169.1	<0.0001
**IL-12p70**	1.00 ± 0.0	ns	15.67 ± 7.33	ns	54.33 ± 4.33	<0.0001
**IL-13**	0.77 ± 0.34	ns	2.24 ± 0.48	ns	2.23 ± 0.24	ns
**IL-15**	0.40 ± 0.10	ns	11.50 ± 1.40	<0.0001	18.30 ± 0.60	<0.0001
**IL-17A**	1.39 ± 0.68	ns	9.50 ± 1.84	0.0004	8.78 ± 0.91	0.0008
**IL-1RA**	1.04 ± 0.06	ns	15.11 ± 0.84	<0.0001	21.60 ± 1.26	<0.0001
**IL-1α**	1.08 ± 0.15	ns	5.28 ± 0.45	ns	10.62 ± 4.17	0.052
**IL-1β**	1.17 ± 0.08	ns	62.70 ± 4.41	0.0009	154.9 ± 17.25	<0.0001
**IL-2**	1.14 ± 0.04	ns	1.40 ± 0.03	<0.0001	1.43 ± 0.02	<0.0001
**IL-3**	1.48 ± 0.06	0.0293	0.95 ± 0.19	ns	0.85 ± 0.03	ns
**IL-4**	27.33 ± 24.85	ns	97.00 ± 34.53	0.0428	188.3 ± 16.67	0.0006
**IL-5**	0.91 ± 0.06	ns	0.91 ± 0.05	ns	1.01 ± 0.06	ns
**IL-6**	1.10 ± 0.03	ns	43.48 ± 3.03	0.0002	152.0 ± 9.54	<0.0001
**IL-7**	1.00 ± 0.0	ns	182.0 ± 34.78	0.0003	301.0 ± 28.69	<0.0001

**IL-8**	3.69 ± 0.08	ns	945.9 ± 63.97	<0.0001	1840 ± 70.28	<0.0001
**IP-10**	6.40 ± 0.56	ns	1627 ± 128.7	<0.0001	1936 ± 85.10	<0.0001
**MCP-1**	1.62 ± 0.13	ns	182.6 ± 7.13	<0.0001	195.5 ± 1.35	<0.0001
**MIP-1α**	323.0 ± 28.50	ns	273600 ± 31290	0.049	1085000 ± 145700	<0.0001
**MIP-1β**	4.16 ± 0.22	ns	2039 ± 44.92	<0.0001	2000 ± 27.51	<0.0001
**TNFα**	1.10 ± 0.05	ns	51.91 ± 3.22	<0.0001	146.2 ± 6.58	<0.0001
**TNFβ**	1.33 ± 0.44	ns	2.97 ± 0.26	0.0016	3.79 ± 0.08	<0.0001
**VEGF**	0.99 ± 0.07	ns	2.20 ± 0.22	<0.0001	2.09 ± 0.06	0.0001

Statistical analysis was conducted by one-way ANOVA with Tukey's correction for multiple comparisons.

Supplemental Movie Legends

Supplemental Video 1. Live video of *L. crispatus* bEV (red) uptake by endocervical cells (green) from 30 minutes-1 hour of exposure.

Supplemental Video 2. Live video of *L. crispatus* bEV (red) uptake by ectocervical cells (green) from 30 minutes-1 hour of exposure.

Supplemental Video 3. Live video of *L. crispatus* bEV (red) uptake by vaginal epithelial cells (green) from 30 minutes-1 hour of exposure.

Supplemental Video 4. Live video of *G. vaginalis* bEV (red) uptake by endocervical cells (green) from 30 minutes-1 hour of exposure.

Supplemental Video 5. Live video of *G. vaginalis* bEV (red) uptake by ectocervical cells (green) from 30 minutes-1 hour of exposure.

Supplemental Video 6. Live video of *G. vaginalis* bEV (red) uptake by vaginal epithelial cells (green) from 30 minutes-1 hour of exposure.

Supplemental Video 7. Live video of *M. mulieris* bEV (red) uptake by endocervical cells (green) from 30 minutes-1 hour of exposure.

Supplemental Video 8. Live video of *M. mulieris* bEV (red) uptake by ectocervical cells (green) from 30 minutes-1 hour of exposure.

Supplemental Video 9. Live video of *M. mulieris* bEV (red) uptake by vaginal epithelial cells (green) from 30 minutes-1 hour of exposure.

Supplemental Video 10. Live video of NYC control bEV isolates (red) uptake by endocervical cells (green) from 30 minutes-1 hour of exposure.

Supplemental Video 11. Live video of NYC control bEV isolates (red) uptake by ectocervical cells (green) from 30 minutes-1 hour of exposure.

Supplemental Video 12. Live video of NYC control bEV isolates (red) uptake by vaginal epithelial cells (green) from 30 minutes-1 hour of exposure.
